# Author Correction: Targeting chondrocytes for arresting bony fusion in ankylosing spondylitis

**DOI:** 10.1038/s41467-025-56680-6

**Published:** 2025-02-07

**Authors:** Fenli Shao, Qianqian Liu, Yuyu Zhu, Zhidan Fan, Wenjun Chen, Shijia Liu, Xiaohui Li, Wenjie Guo, Gen-Sheng Feng, Haiguo Yu, Qiang Xu, Yang Sun

**Affiliations:** 1 State Key Laboratory of Pharmaceutical Biotechnology, Department of Biotechnology and Pharmaceutical Sciences, School of Life Sciences, Nanjing University, 163 Xianlin Avenue, Nanjing, 210023 China; 2 College of Pharmacy, Nanjing University of Chinese Medicine, 138 Xianlin Avenue, Nanjing, 210023 China; 3 Department of Rheumatology and Immunology, Children’s Hospital of Nanjing Medical University, 72 Guangzhou Road, Nanjing, 210008 China; 4 Affiliated Hospital of Nanjing University of Chinese Medicine, 155 Hanzhong Road, Nanjing, 210029 China; 5 Department of Radiology, Children’s Hospital of Nanjing Medical University, 72 Guangzhou Road, Nanjing, 210008 China; 6 Department of Pathology, and Division of Biological Sciences, University of California San Diego, La Jolla, CA 92093 USA; 7 Jiangsu Key Laboratory of New Drug Research and Clinical Pharmacy, Xuzhou Medical University, 209 Tongshan Road, Xuzhou, 221004 China; 8 Chemistry and Biomedicine Innovation Center (ChemBIC), Nanjing University, 163 Xianlin Avenue, Nanjing, 210023 China

Correction to: *Nature Communications* 10.1038/s41467-021-26750-6, published online 11 November 2021

In the version of the article initially published, in Fig. 2e, the image for the 7-month Ctrl was inadvertently a duplicate of the 2-month CKO image and has now been corrected in the HTML and PDF versions of the article.

