# Supplementary material for: Targeting chondrocytes for arresting bony fusion in ankylosing spondylitis
Source: Nat Commun. 2021 Nov 11;12:6540. doi: 10.1038/s41467-021-26750-6 (PMC8585952; doi:10.1038/s41467-021-26750-6)
Supplement: Supplementary file 1 — Supplementary Information [file 41467_2021_26750_MOESM1_ESM.pdf]

Supplementary Information for:

Targeting chondrocytes for arresting bony fusion in ankylosing spondylitis

Fenli Shao[1, #], Qianqian Liu[1,#], Yuyu Zhu[1,2,#], Zhidan Fan[3,#], Wenjun Chen[4], Shijia Liu[4], Xiaohui Li[5], Wenjie Guo[1], Gen-Sheng Feng[6], Haiguo Yu[3,*], Qiang Xu[1,*], Yang Sun[1,7,*]

[1]State Key Laboratory of Pharmaceutical Biotechnology, Department of Biotechnology and Pharmaceutical Sciences, School of Life Sciences, Nanjing University, 163 Xianlin Avenue, Nanjing 210023, China.
[2]College of Pharmacy, Nanjing University of Chinese Medicine, 138 Xianlin Avenue, Nanjing 210023, China
[3]Department of Rheumatology and Immunology, Children's Hospital of Nanjing Medical University, 72 Guangzhou Road, Nanjing 210008, China.
[4]Affiliated Hospital of Nanjing University of Chinese Medicine, 155 Hanzhong Road, Nanjing 210029, China.
[5]Department of Radiology, Children's Hospital of Nanjing Medical University, 72 Guangzhou Road, Nanjing 210008, China.
[6]Department of Pathology, and Division of Biological Sciences, University of California San Diego, La Jolla, CA 92093, USA.
[7]Chemistry and Biomedicine Innovation Center (ChemBIC), Nanjing University, 163 Xianlin Avenue, Nanjing 210023, China.

* Corresponding to

Yang Sun, Ph.D., Professor or Qiang Xu, Ph.D.,Professor

School of Life Sciences, Nanjing University, Nanjing 210023 China

E-mail: yangsun@nju.edu.cn (Y. Sun) or qiangxu@nju.edu.cn (Q. Xu)

Haiguo Yu, M.D., Professor

Department of Rheumatology and Immunology, Children's Hospital of Nanjing Medical University, Nanjing, 210008 China

E-mail: haiguoyu2000@163.com (H. Yu)

Contents:

1. Supplementary Figures 1-29;
2. Supplementary Table 1;
3. Title and captions for Supplementary Movie 1-4.

Supplementary Figures

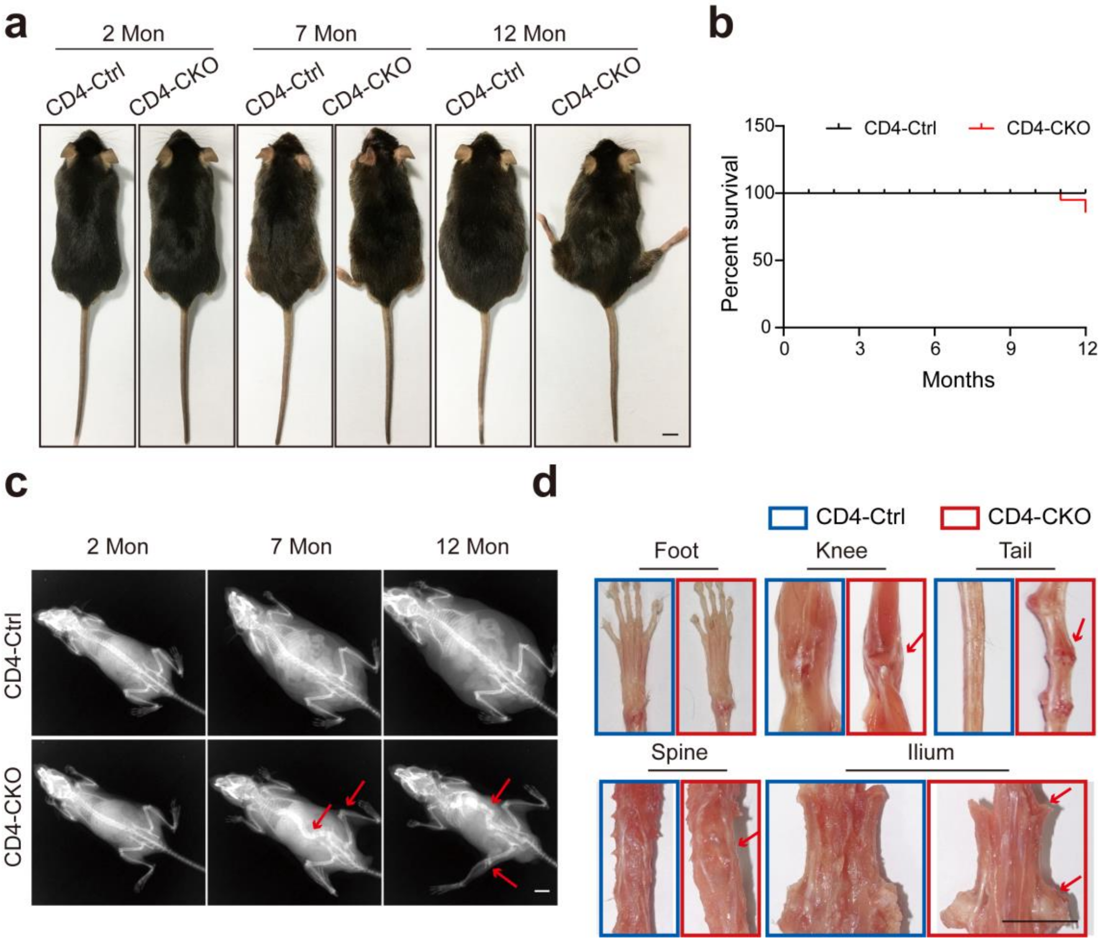

Supplementary Fig. 1. **Conditional deletion of SHP2 mediated by CD4-Cre induces AS-like bone disease.** (a) Gross images of male mice. The images show skeleton abnormalities in mature and aged male CD4-CKO mice. (b) The mortality rate of female CD4-Ctrl and CD4-CKO mice (*n* = 10) (c) Representative X-ray images indicate scoliosis, kyphosis (red arrows) and pathological new bone formation in mature and aged CD4-CKO mice. (d) Photographs of 12-month-old mice show bone deformity (red arrows) in spine, knee and hip joints, rather than ankles and feet of aged CD4-CKO mice and skeleton of CD4-Ctrl mice. (a, c, d) Scale bars: 1 cm. Data are representative of three independent biological replicates.

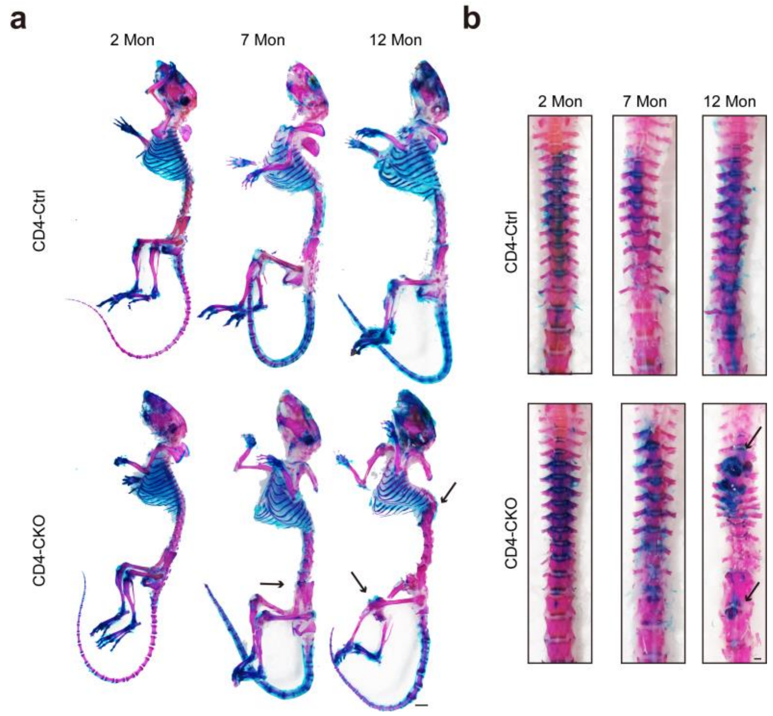

Supplementary Fig. 2. Whole skeletons show bone deformation in mature and aged CD4-CKO mice. Whole skeletons of CD4-CKO mice and CD4-Ctrl littermates were stained with alizarin red (bone) and alcian blue staining (cartilage). (a) Representative whole skeleton images of mice show kyphosis and ankylosis of spine, hip and knee joints. Scale bar：0.5 cm. (b) Photographs of chondroma in spine of aged CD4-CKO mice as black arrows indicated. Scale bar: 0.1 cm. (a, b) Data are representative of three independent biological replicates.

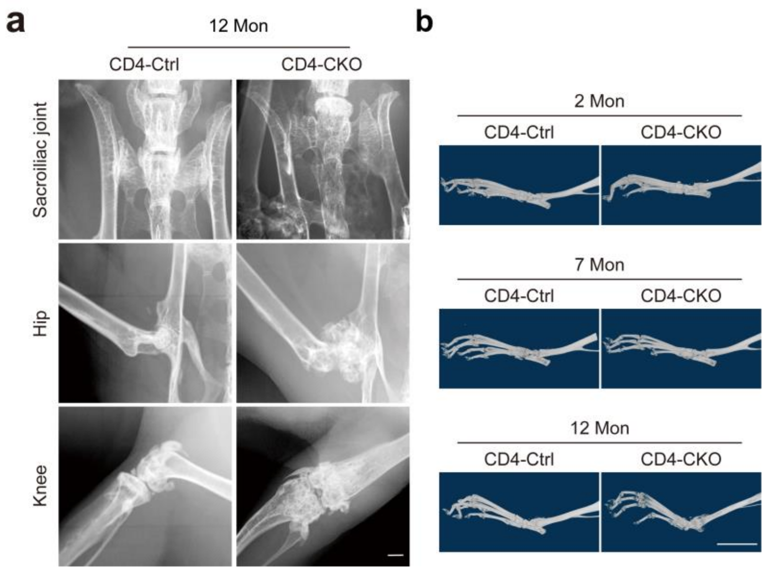

Supplementary Fig. 3. **Pathological new bone formation in axial joints, rather than feet.** (a) Representative X-ray images indicate fusion of sacroiliac, hip and knee joints. (b) Representative μ-CT radiographs show natural structure of feet in CD4-Ctrl and CD4-CKO mice. (a, b) Scale bars: 1 mm. Data are representative of three independent biological replicates.

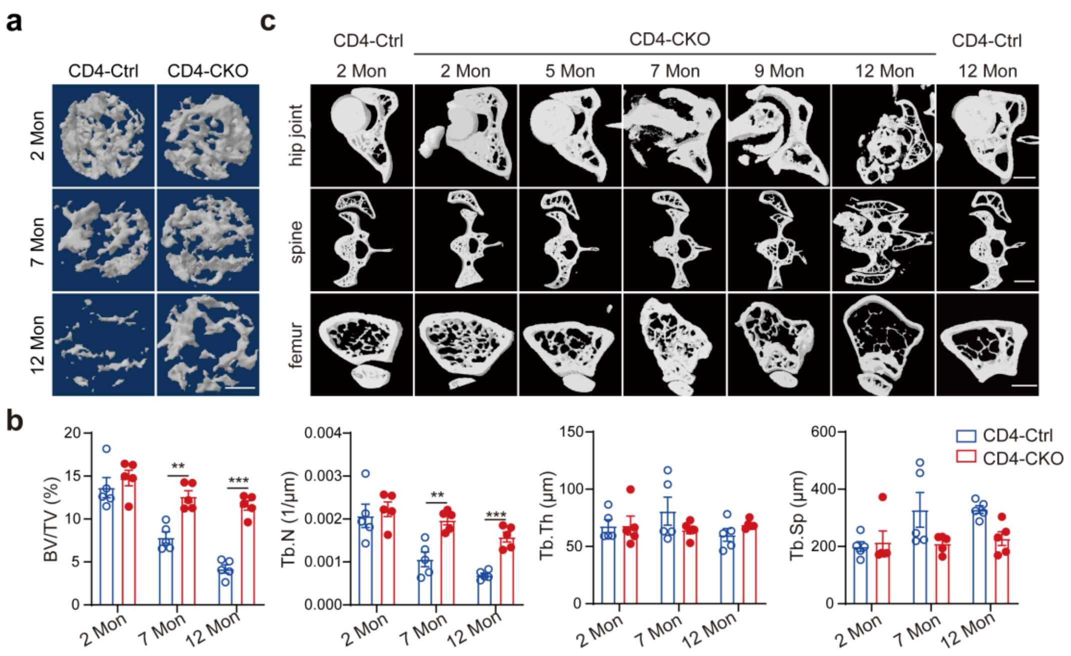

Supplementary Fig. 4. Cortical bone and subchondral bone loss in aged CD4-CKO mice. (a-b) Trabecular bones of distal femurs isolated from CD4-Ctrl and CD4-CKO mice were reconstructed and measured. 3D μ-CT images (a) and parameters (b) show increased trabecular bones in femurs of mature and aged CD4-CKO mice. (a) Scale bars: 0.5 mm. (b) Bone volume per tissue volume (BV/TV), trabecular number (Tb.N), trabecular thickness (Tb.Th) and trabecular spacing (Tb.Sp) (n = 5). Data are presented as mean ± SEM. $^{**}p < 0.01$, $^{***}p < 0.001$, determined by two-tailed Student's *t*-test. (c) Transverse sections of joints show bone deformation and loss of cortical bone in aged CD4-CKO mice as compared with young and aged CD4-Ctrl mice. Scale bars:1 mm. (a, c) Data are representative of three independent biological replicates.

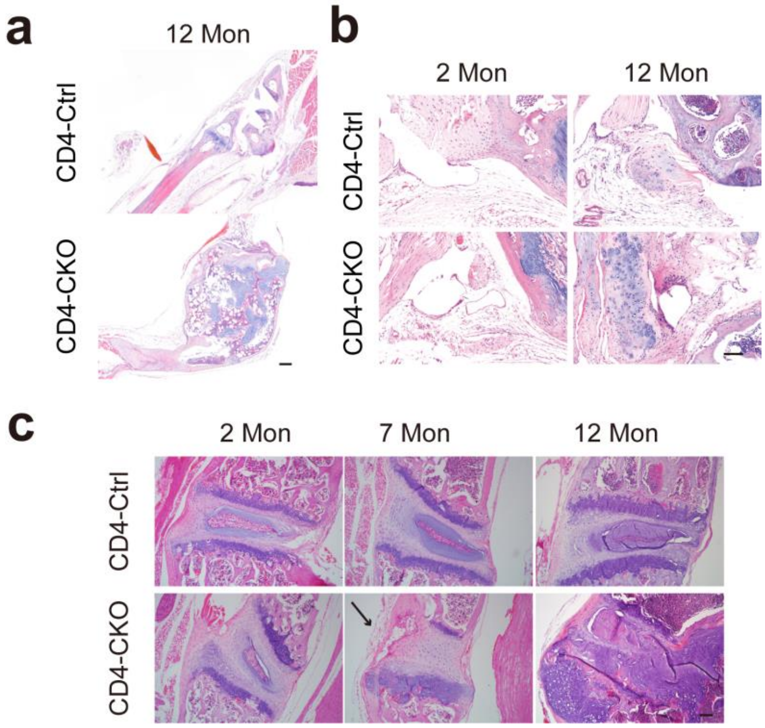

Supplementary Fig. 5. **Histological analysis shows bone lesions in mature and aged CD4-CKO mice.** (a) H&E staining images show natural structure of wrist from CD4-Ctrl mice and bone deformation and cartilage disorder in wrist of CD4-CKO mice. Scale bars: 200 μm. (b) H&E staining images of articular cavity of knee joints indicate natural structure of CD4-Ctrl mice and young CD4-CKO mice and cartilage and bone disorder in articular cavity of aged CD4-CKO mice. Scale bars: 100 μm. (c) Histological images of spines show inflammation (black arrow) and synovial hyperplasia in mature CD4-CKO mice and cartilage and bone disorder in aged CD4-CKO mice. Scale bars: 100 μm. (a-c) Data are representative of three independent biological replicates.

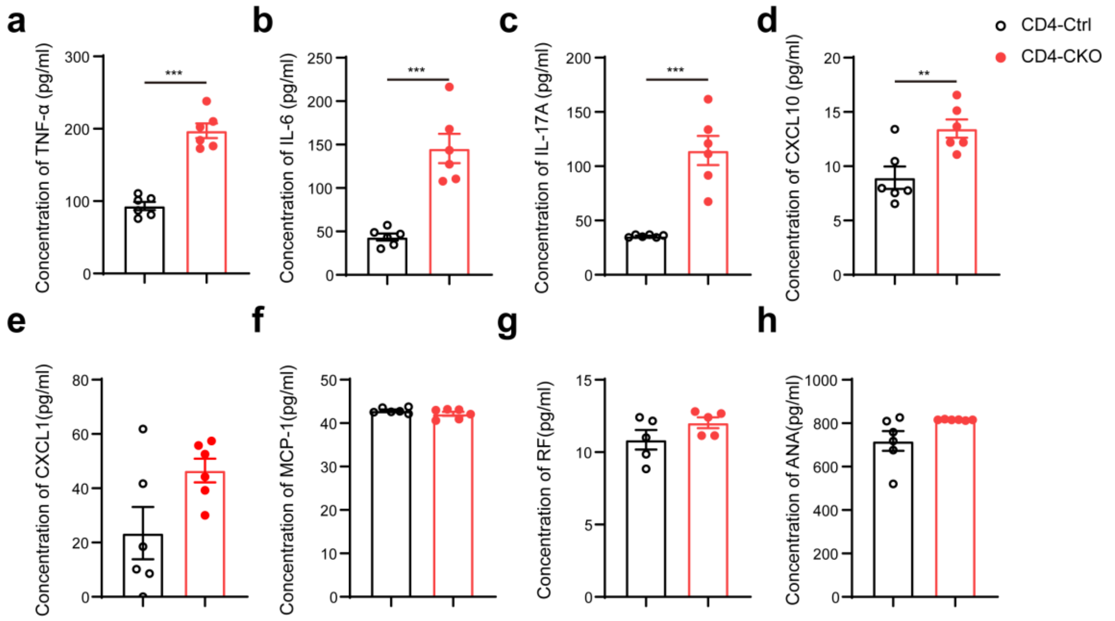

Supplementary Fig. 6. Increased inflammatory cytokines in serum of aged CD4-CKO mice. Serum of 12-month-old mice CD4-Ctrl and CD4-CKO mice were collected and the inflammation cytokines were measured by ELISA. (a) Tumor necrosis factor-α (TNF-α). (b) Interleukin-6 (IL-6). (c) Interleukin-17A (IL-17A). (d) Interferon γ–induced protein-10 (IP-10, also known as CXCL10). (e) C-X-C motif chemokine ligand 1 (CXCL1). (f) Monocyte chemoattractant protein-1 (MCP-1). (g) Rheumatoid factor (RF). (h) Antinuclear antibody (ANA). (a-h) (n = 6). Data are presented as mean ± SEM. *p < 0.05, **p < 0.01, ***p < 0.001, determined by two-tailed Student's t-test.

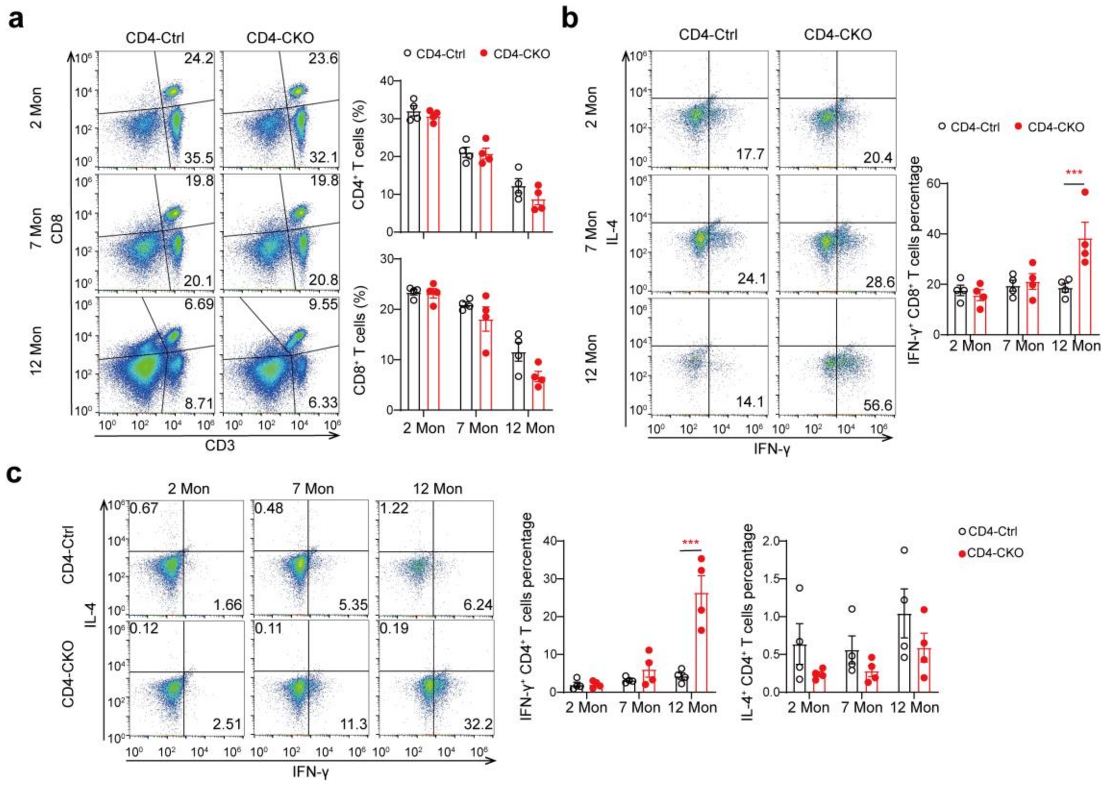

Supplementary Fig. 7. **Increased IFN-γ^+ T cells in lymph nodes of aged CD4-CKO mice.** Flow cytometry analyzed the T cells subsets in the inguinal lymph nodes of CD4-Ctrl and CD4-CKO mice at indicated ages. (a) The proportion of $CD3^+CD4^+$ T cells and $CD3^+CD8^+$ T cells were comparable in CD4-Ctrl and Age-matched CD4-CKO mice (n = 4). (b) Flow cytometry analysis of $CD8^+$ T cells indicates similar proportion of IFN-$\gamma^+$$CD8^+$ T cell in young and mature CD4-Ctrl mice and CD4-CKO mice and more IFN-$\gamma^+$$CD8^+$ T cells in lymph nodes of aged CD4-CKO mice than age-matched CD4-Ctrl mice (n = 4). (c) Flow cytometry analysis of IFN-$\gamma^+$$CD4^+$ Th1 cells and IL-$4^+$$CD4^+$ Th2 cells shows comparable Th1 and Th2 cells in young and mature CD4-Ctrl and CD4-CKO mice, and increased Th1 cells in lymph nodes of aged CD4-CKO mice as compared with age-matched CD4-Ctrl mice (n = 4). (a-c) Data are presented as mean ± SEM. *$p < 0.05$, **$p < 0.01$, ***$p < 0.001$, determined by two-tailed Student's t-test.

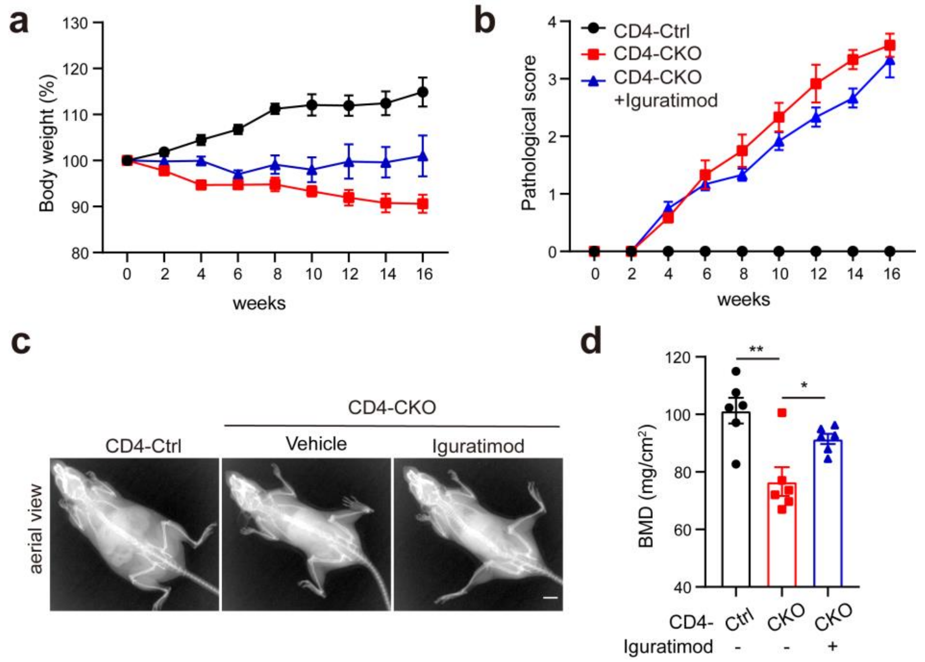

Supplementary Fig. 8. Anti-rheumatic drug Iguratimod does not improve AS-like bone disease in CD4-CKO mice. 6-month-old CD4-CKO mice were treatment with 100 mg/kg Iguratimod and the body weights and pathological scores were monitored for 16 weeks. (a) Body weights of CD4-Ctrl and CD4-CKO mice treated with Iguratimod or not. (b) Pathological scores of bone disease in CD4-Ctrl, CD4-CKO mice and CD4-CKO mice treated with Iguratimod. (c-d) Representative X-ray images (c) and femoral BMD. (d) of CD4-CKO mice treated with Iguratimod for 16 weeks or not and CD4-Ctrl littermates. (c) Scale bar: 1 cm. (a, b, d) (n = 6). Data are presented as mean ± SEM. *p < 0.05, **p < 0.01, ***p < 0.001, determined by two-tailed Student's t-test. (c) Data are representative of three independent biological replicates.

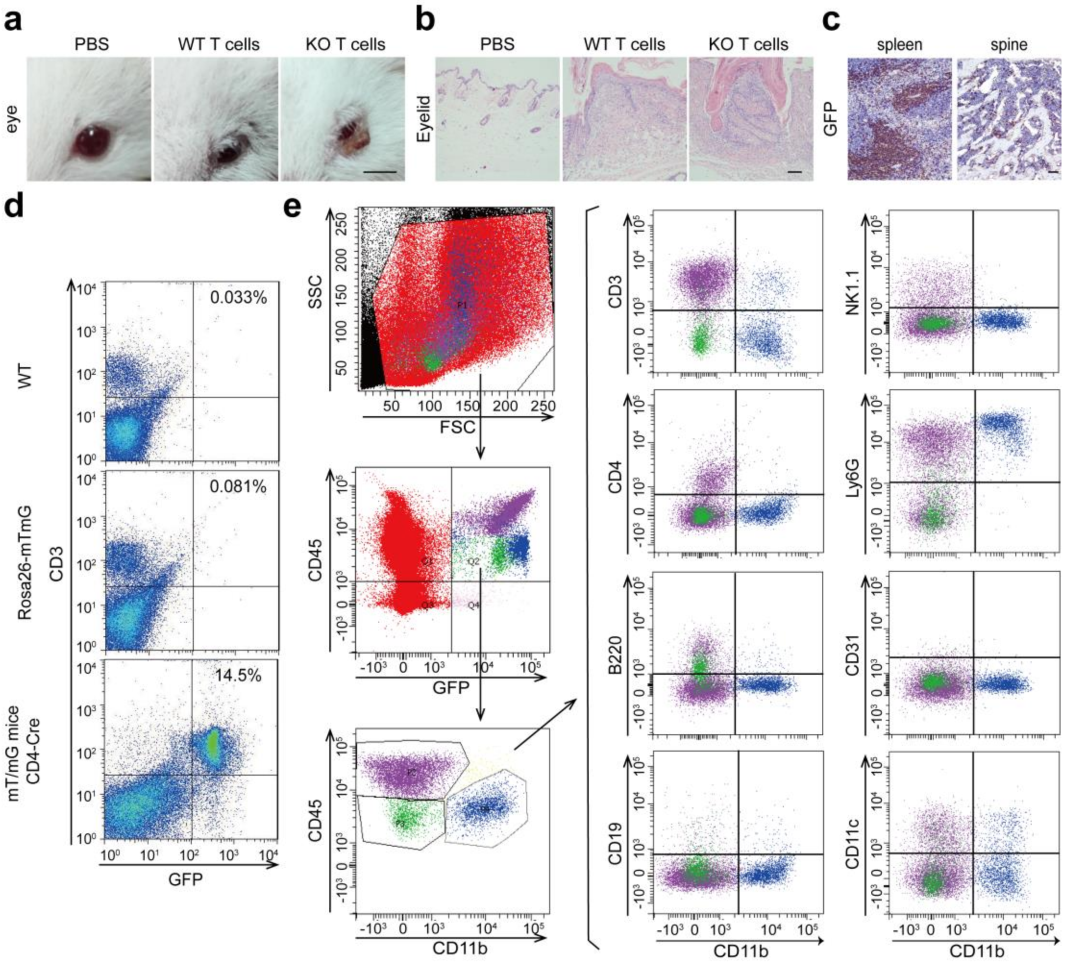

Supplementary Fig. 9. **CD4-Cre mediates SHP2 deletion in several subsets of bone marrow of CD4-CKO;Rosa26-mTmG mice.** (a) Photographs of NCG mice show WT and KO CD3$^+$T cells induced eyelid dermatitis in NCG mice. Scale bar: 0.5 cm. (b) Histological analysis of eyelid in NCG mice shows inflammatory cells infiltration in NCG mice transferred with WT or KO CD3$^+$ T cells as compared with normal NCG mice. Scale bars: 100 µm. (c) GFP immunostaining images show GFP$^+$ cells in spleen and GFP$^+$ cells in bone marrow from young CD4-CKO;Rosa26-mTmG mice. Scale bars: 50 µm. (d) Flow cytometry analyses indicates CD3$^+$ T cells of spleen from CD4-CKO;Rosa26-mTmG mice were GFP$^+$ cells. (e) Bone marrow cells of young CD4-CKO;Rosa26-mTmG mice were analyzed by flow cytometry and three GFP$^+$ subsets were detected in the bone marrow, including the green subset (CD45$^+$CD3$^-$CD4$^-$CD11b$^-$CD19$^-$NK1.1$^-$CD11c$^-$ cells), purple subset (CD45$^+$CD3$^+$ cells) and blue subset (CD45$^+$CD11b$^+$Ly6G$^+$ cells). (a-e) Representative results of three independent experiments were shown.

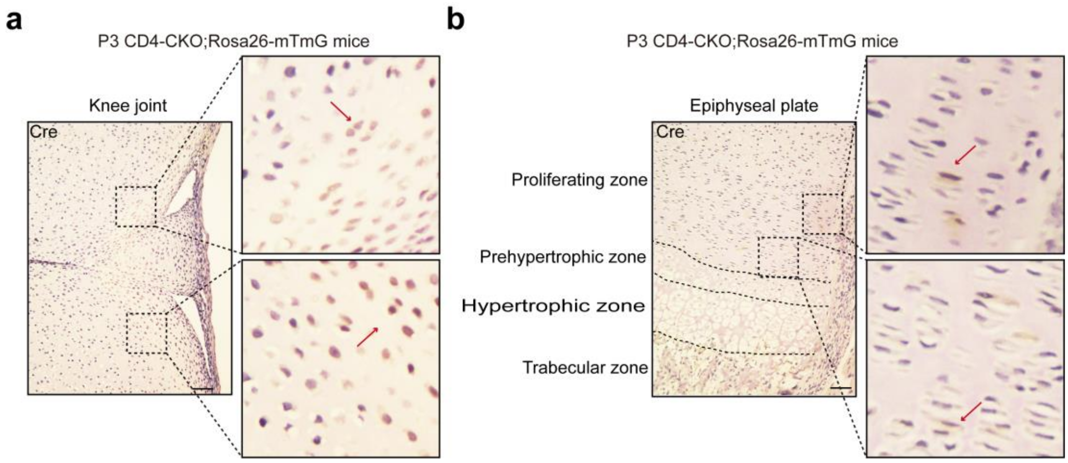

Supplementary Fig. 10. **CD4-Cre mediates SHP2 deficiency in proliferating chondrocytes.** (a-b) Immunohistochemistry analysis of Cre expression knee joint (a) and femoral growth plate (b) show Cre expressed in proliferating chondrocytes (red arrows). Scale bars:100 μm. Data are representative of three independent experiments.

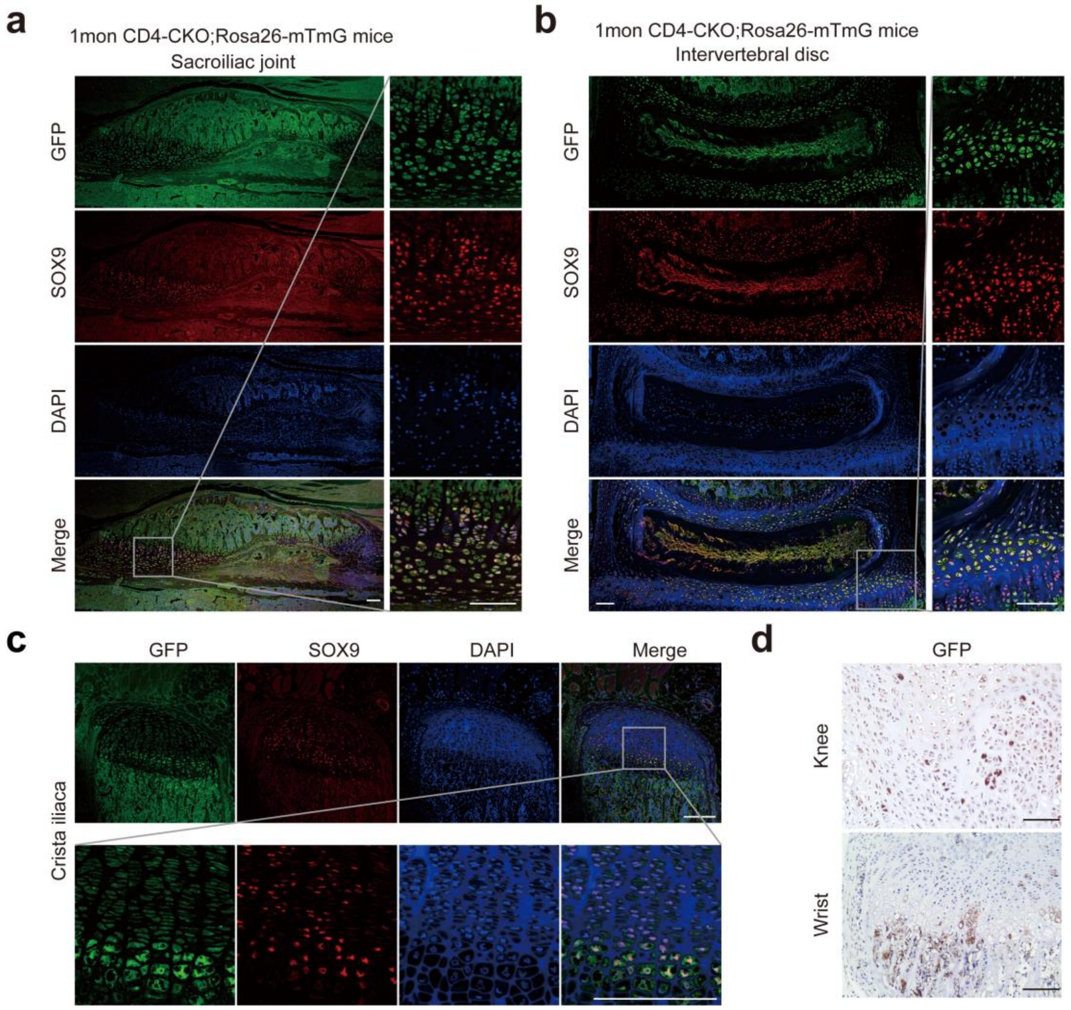

Supplementary Fig. 11. GFP⁺ cells are pre-hypertrophic and hypertrophic chondrocytes in the joints of young CD4-CKO;Rosa26-mTmG mice. The sections of joints obtained from 1-month-old CD4-CKO;Rosa26-mTmG mice were immunostained with marker protein GFP, transcriptional regulator SOX9 and nuclear counterstain DAPI. (a-c) The Immunofluorescence staining images of sacroiliac joint (a), intervertebral disc (b), and crista iliaca (c) show GFP^+ pre-hypertrophic and hypertrophic chondrocytes in the joints. (d) The immunohistochemistry images show GFP^+ chondrocytes in wrist and knee joint of 1-month-old CD4-CKO mice. Scale bars: 100 μm. Data are representative of three independent experiments.

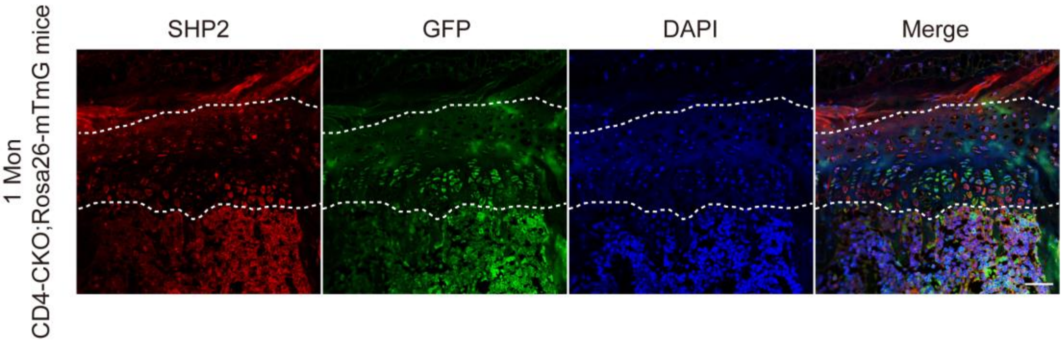

Supplementary Fig. 12. **GFP$^+$ pre-hypertrophic and hypertrophic chondrocytes are SHP2-deficient in CD4-CKO;Rosa26-mTmG mice.** Spine section of 1-month-old CD4-Cre;Rosa26-mTmG mice were immunostained with marker protein GFP, SHP2 and nuclear counterstain DAPI. GFP and SHP2 were expressed in different subtype of chondrocytes in the cartilaginous endplate of spine in 1-month-old CD4-Cre;Rosa26-mTmG mice. The area inside the two white dotted lines is cartilaginous endplate. Scale bar: 50 μm. Data are representative of three independent experiments.

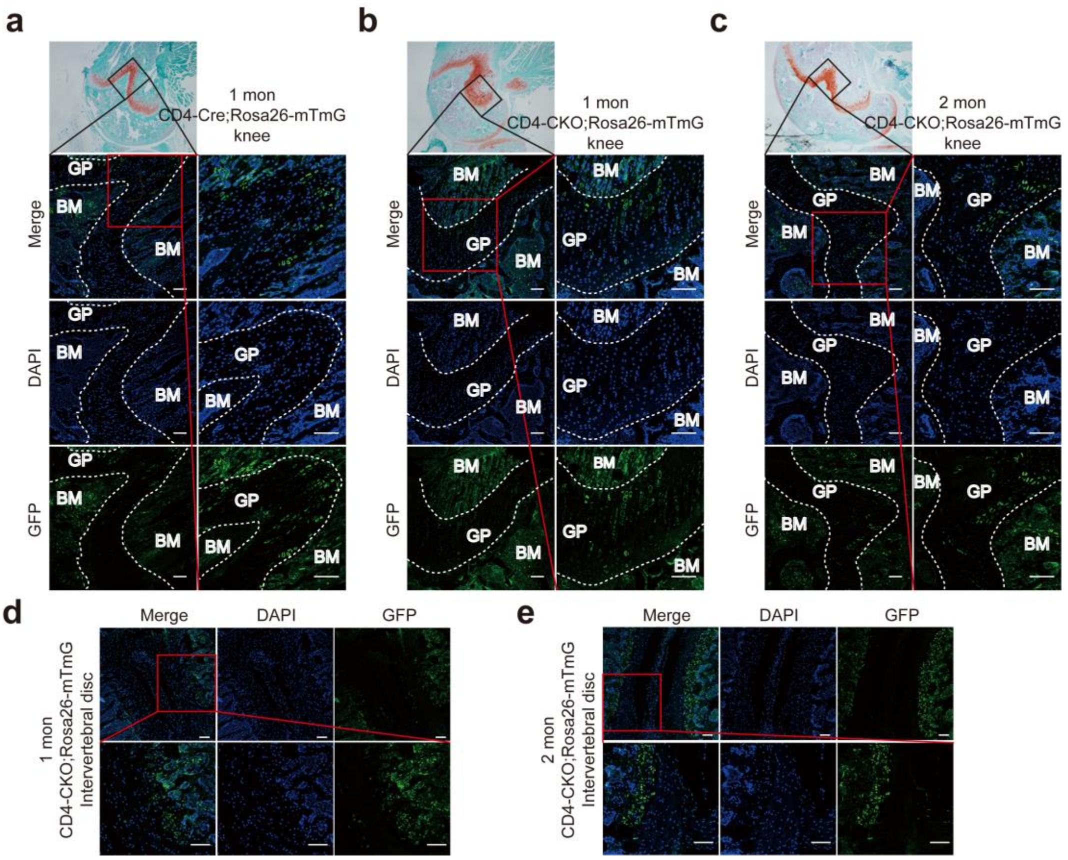

Supplementary Fig. 13. **Similar proportion of GFP$^+$ differentiated chondrocytes in CD4-Cre;Rosa26-mTmG mice and age-matched CD4-CKO;Rosa26-mTmG mice.** Joint sections obtained from CD4-Cre;Rosa26-mTmG mice and CD4-CKO;Rosa26-mTmG mice at indicated ages and immunostained with marker protein GFP and nuclear counterstain DAPI. (a-c) Partial pre-hypertrophic and hypertrophic chondrocytes in the growth plate of 1-month-old CD4-Cre;Rosa26-mTmG mice (a), 1-month-old CD4-CKO;Rosa26-mTmG mice (b) and 2-month-old CD4-CKO;Rosa26-mTmG mice (c) were GFP$^+$ cells. BM, bone marrow; GP, growth plate. (d-e) Differentiated chondrocytes in cartilaginous endplate from 1-month-old CD4-CKO;Rosa26-mTmG mice (d) and 2-month-old CD4-CKO;Rosa26-mTmG mice (e) were GFP$^+$ cells. (a-e) Scale bars: 100 μm. Data are representative of three independent experiments.

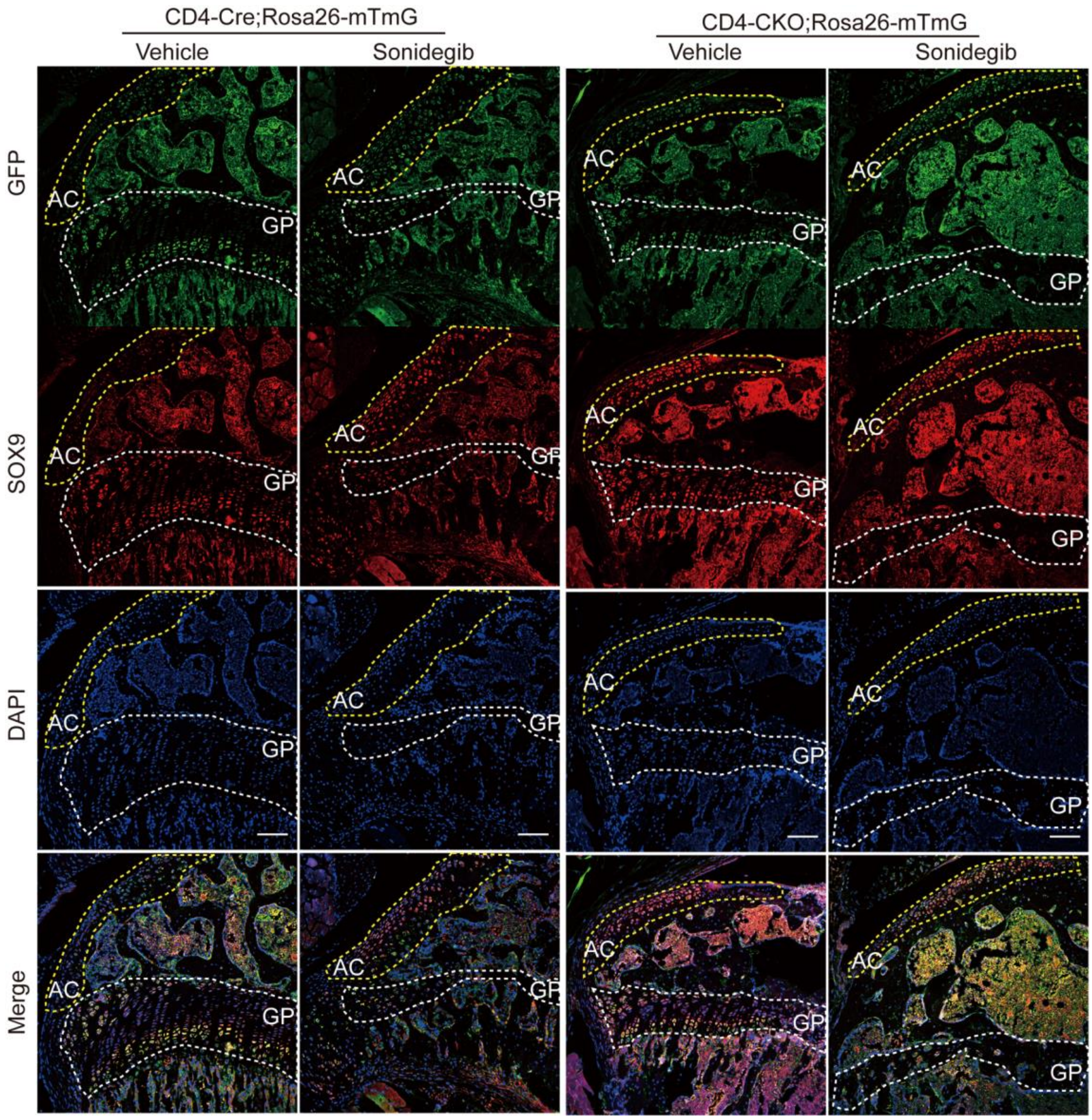

Supplementary Fig. 14. **Sonidegib abolishes the growth plate in mice.** 3-week-old CD4-CKO;Rosa26-mTmG mice and CD4-Cre;Rosa26-mTmG littermates were orally gavaged every other day with Smo inhibitor, sonidegib (50 mg/kg) or vehicle control (0.5% methylcellulose) for 3 times. Femoral sections from 2-month-old mice were immunostained with marker protein GFP, transcriptional regulator SOX9 and nuclear counterstain DAPI. Representative Immunofluorescence images show loss of $SOX9^+$ chondrocytes and growth plate in CD4-Ctrl and CD4-CKO mice treated with Sonidegib. AC, articular cartilage; GP, growth plate. Scale bars: 100 µm. Data are representative of three independent experiments.

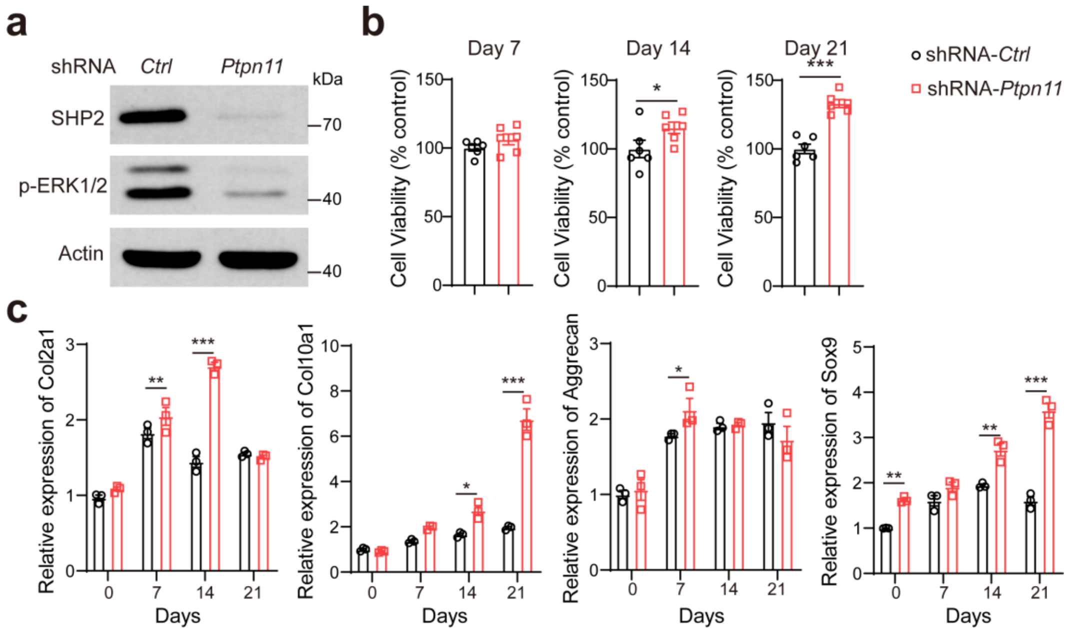

Supplementary Fig. 15. SHP2 deficiency promotes the proliferation and differentiation of ATDC5 cells. (a) Mouse chondrogenic cell line ATDC5 cells were transfected with shRNA-*Ctrl* and shRNA-*Ptpn11* lentivirus respectively and the SHP2 expression were analyzed by Western blot. The results show decreased p-ERK1/2 level in ATDC5 cells with knock down of SHP2. Data are representative of three independent biological replicates. (b-c) ATDC5 cells were differentiated in medium with ITS and the proliferation and differentiation were analyzed by MTT activity (n = 6) (b) and qPCR (n = 3) (c) respectively. The proliferation activity and expression of gene *Col2a1*, *Col10a1*, *Aggrecan* and *Sox9* were increased in ATDC5 cells transfected with shRNA-*Ptpn11* lentivirus. Data are presented as mean ± SEM. *$p < 0.05$, **$p < 0.01$, ***$p < 0.001$, determined by two-tailed Student's t-test.

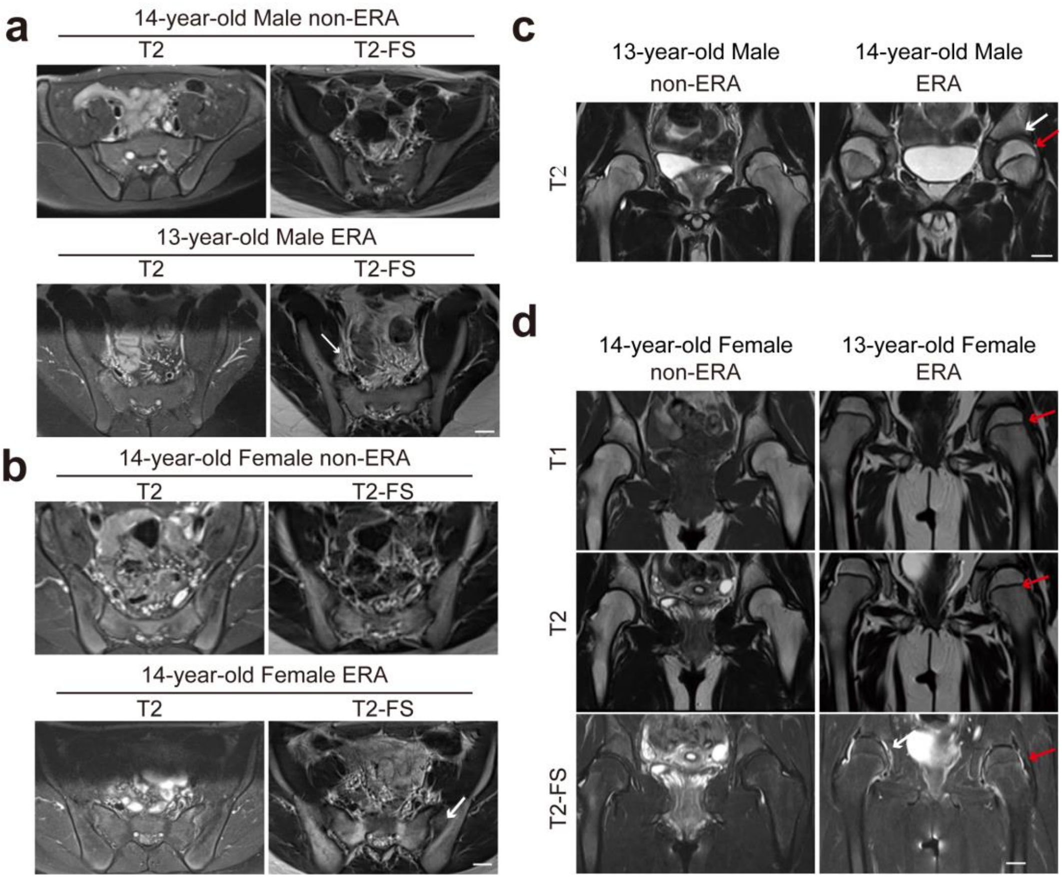

Supplementary Fig. 16. Increased thickness of growth plate in joints of ERA patients. MRI with T1 weighed, T2 weighed and T2 weighed fat saturated (T2-FS) sequence of patients with non-ERA or ERA. Sacroiliac joints (a-b) and Hip joints (c-d) of patients show arthritis, bone deformation (white arrows) and thicker growth plate (red arrows) in joints of ERA patients than those with non-ERA. Scale bars: 2.5 cm. Data are representative of three independent biological replicates.

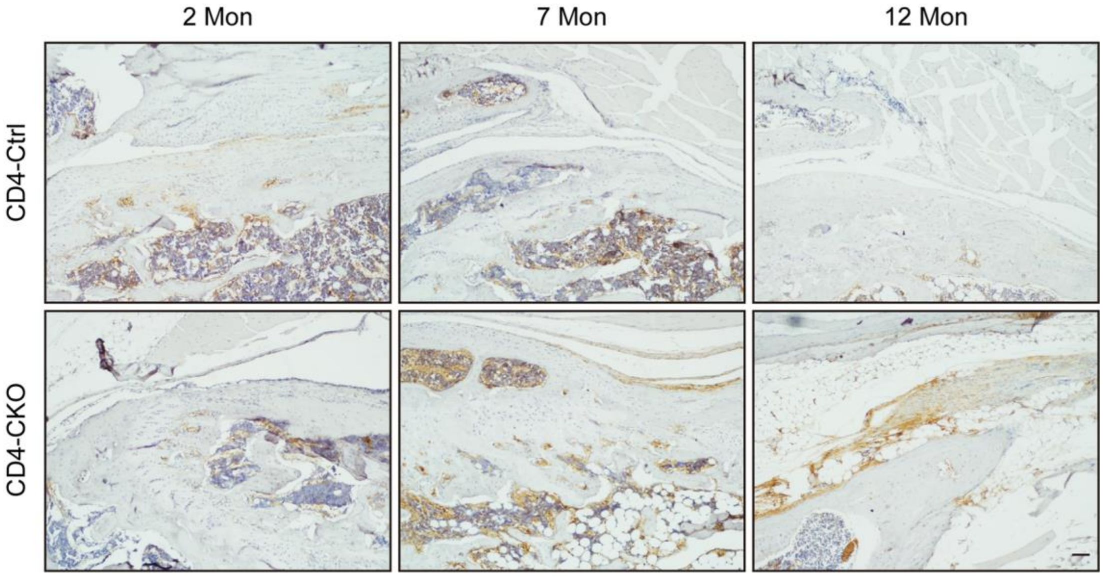

Supplementary Fig. 17. Increased pSmad1/5 levels in enthesis of mature and aged CD4-CKO mice. The immunohistochemistry analysis of pSmad1/5 levels in enthesis of knee joint. Scale bar: 100 μm. Data are representative of three independent experiments.

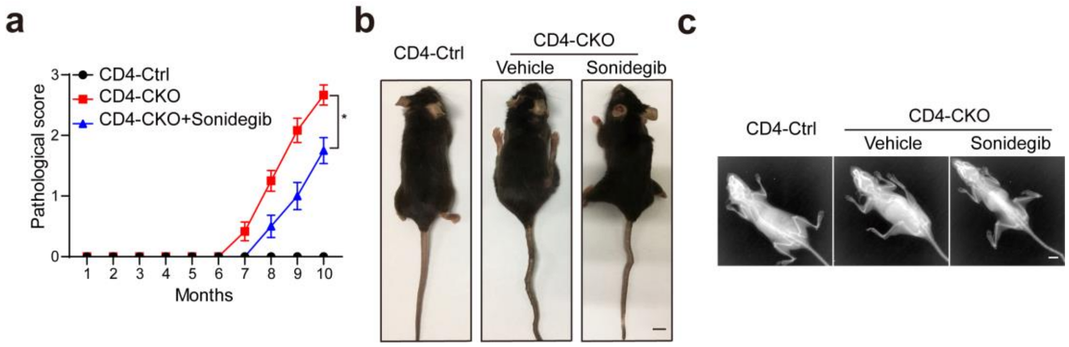

Supplementary Fig. 18. Treatment of sonidegib at 6-month-old attenuates AS-like bone disease in CD4-CKO mice. 6-month-old CD4-CKO mice and CD4-Ctrl littermates were gavaged daily with the Smo inhibitor, sonidegib (50 mg/kg) or vehicle control (0.5% methylcellulose) for 4 months. (a) Pathological scores show treatment of sonidegib alleviated bone disease in CD4-CKO mice (n = 6). Data arepresented as mean ± SEM. *p < 0.05, determined by two-tailed Student's t-test. (b-c) Gross images and radiographic images of 10-month-old mice show alleviative bone lesions in CD4-CKO mice treated with sonidegib as compared with CD4-CKO mice treated with vehicle. Scale bars: 1 cm. Data are representative of three independent biological replicates.

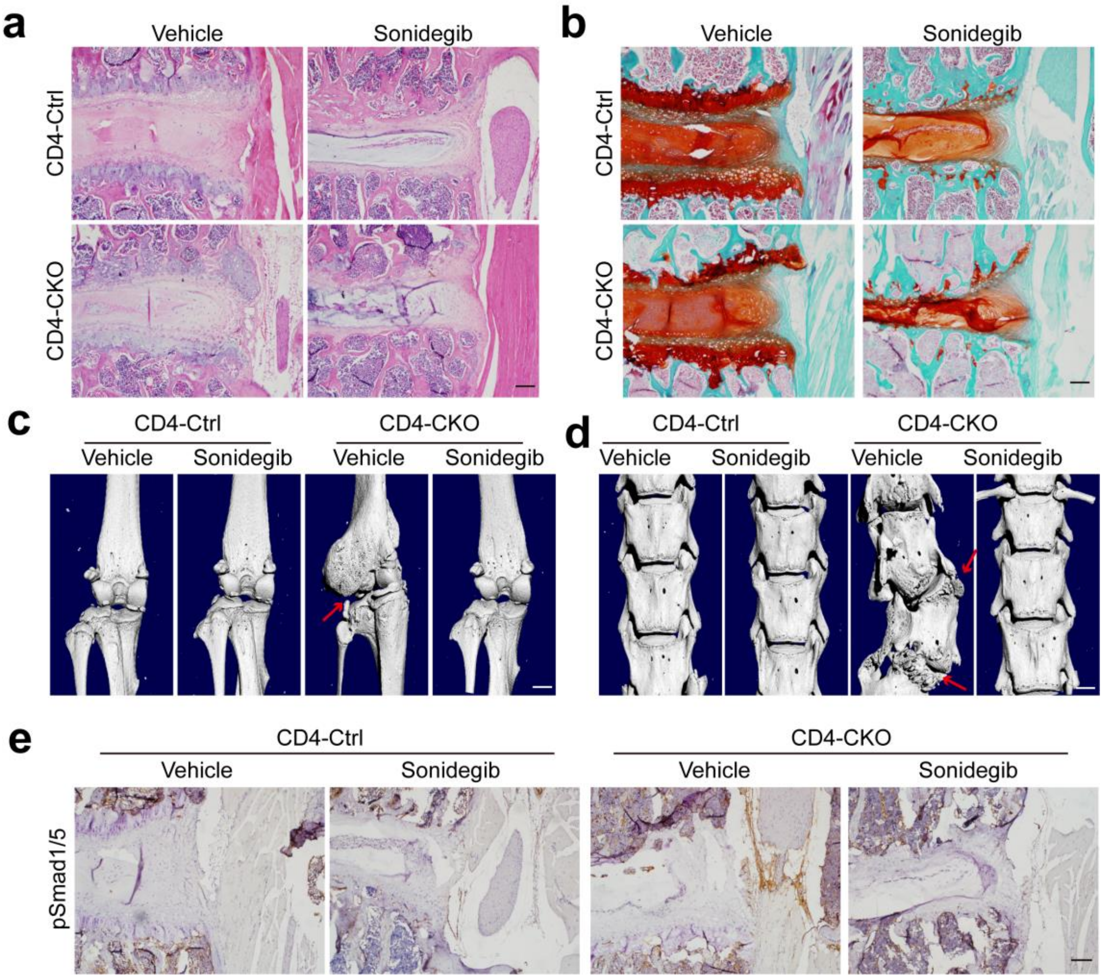

Supplementary Fig. 19. Sonidegib alleviates AS-like bone disease in CD4-CKO mice. 7-month-old CD4-CKO mice and CD4-Cre littermates were orally gavaged with Smo inhibitor, sonidegib (100 mg/kg) for 4 months. (a) H&E staining of spine sections. (b) SOFG staining of spine sections. (c and d) μ-CT radiographs of knee joint (c) and spine (d) of 11-month-old mice. Arrows show ectopic new bone formation. (e) Immunohistochemical staining of pSmad1/5 protein levels in spine of 11-month-old mice. (a, b, e) Scale bars: 100 μm. (c, d) Scale bars: 2.5 cm. Data are representative of three independent biological replicates.

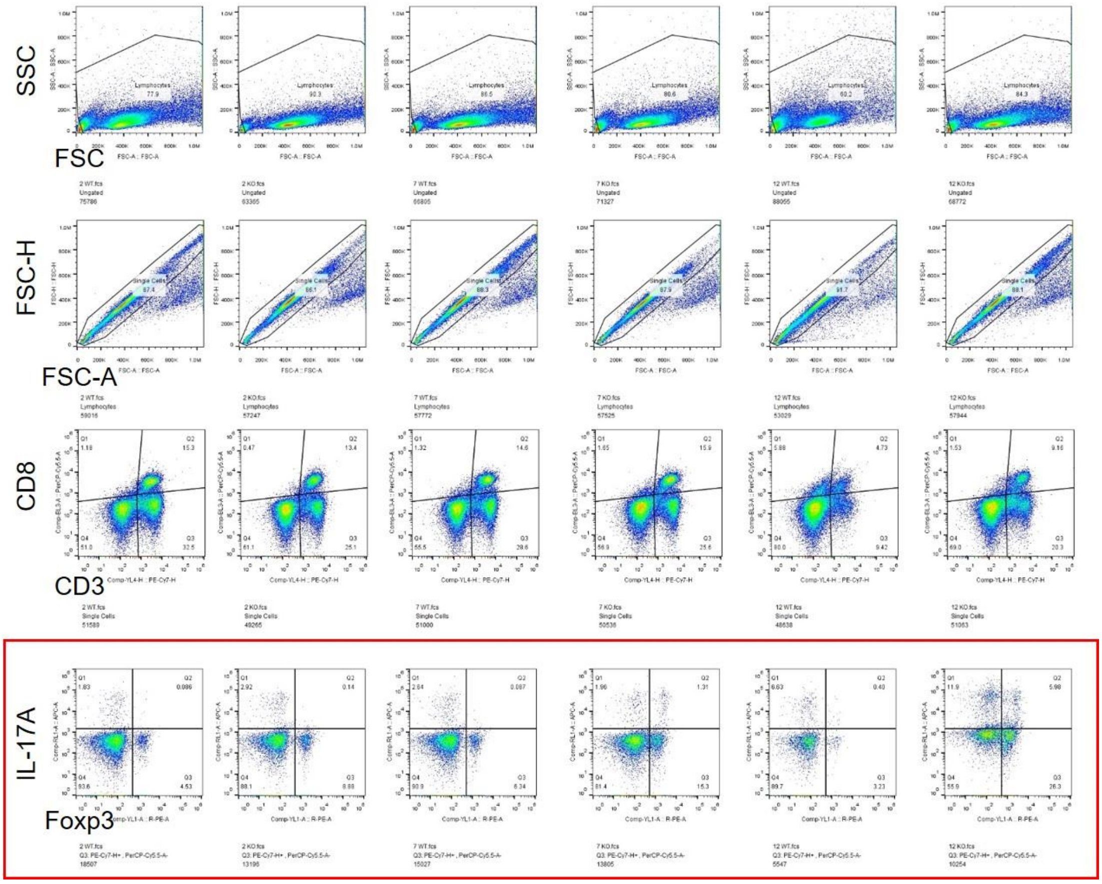

Supplementary Fig. 20. Gating strategies for Fig.3a.

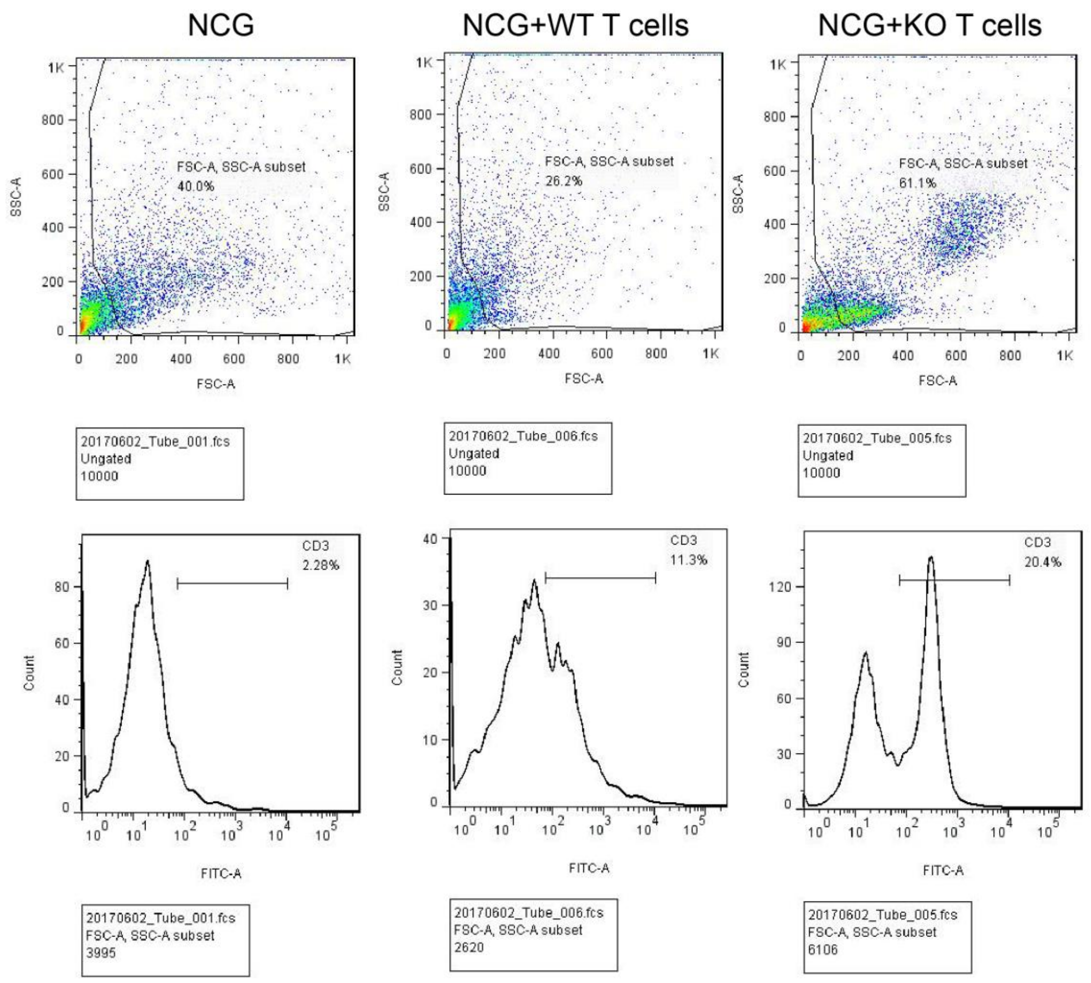

Supplementary Fig. 21. Gating strategies for Fig. 3b.

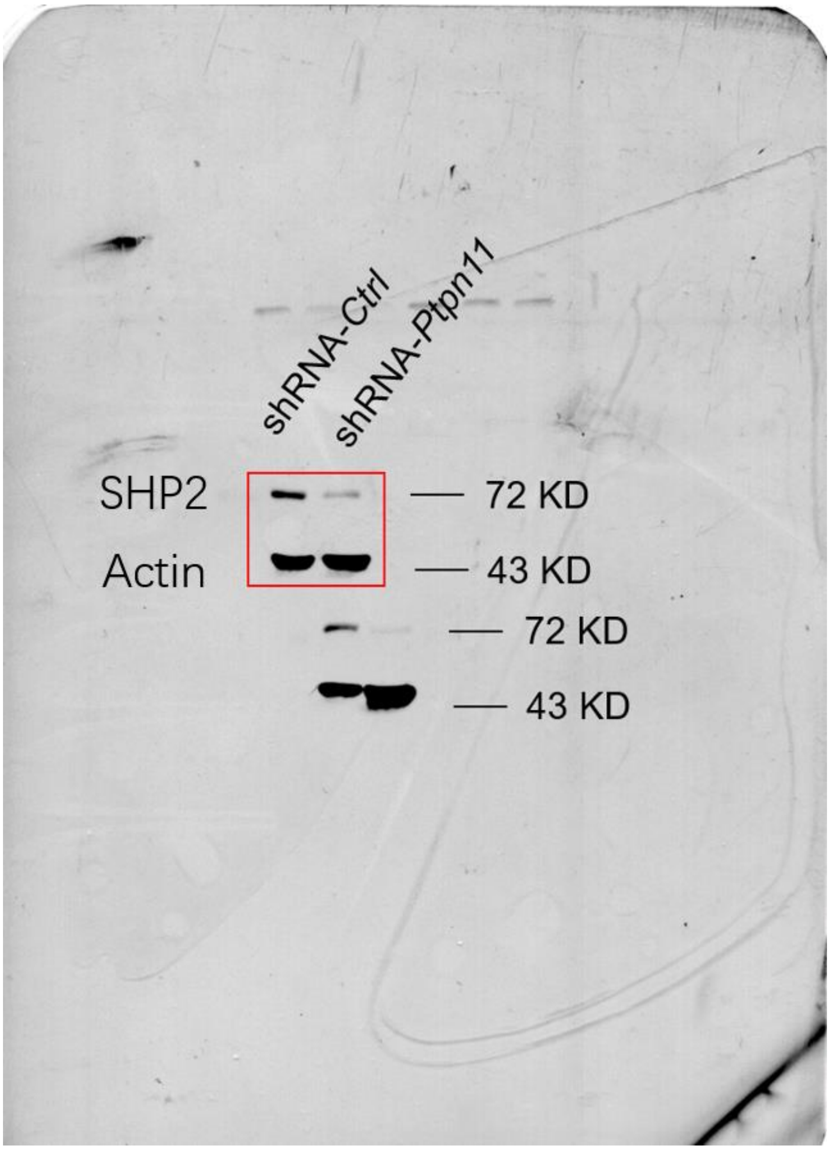

Supplementary Fig. 22. Uncropped scans of the Western blots shown in Fig. 6d.

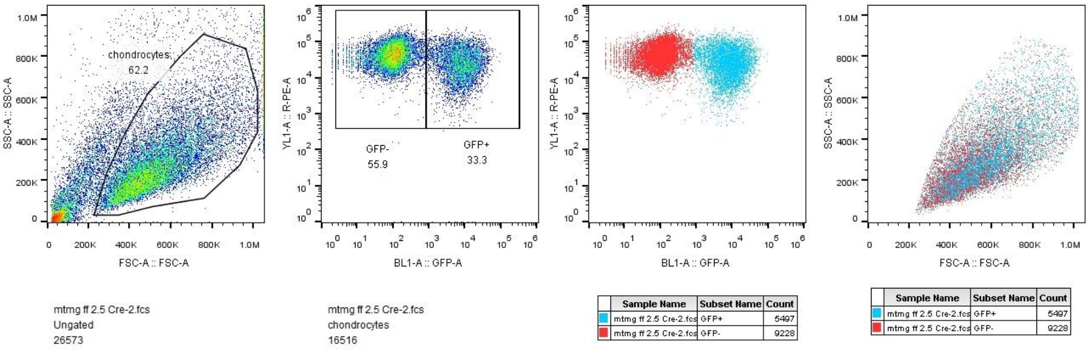

Supplementary Fig. 23. Gating strategies for Fig. 6g.

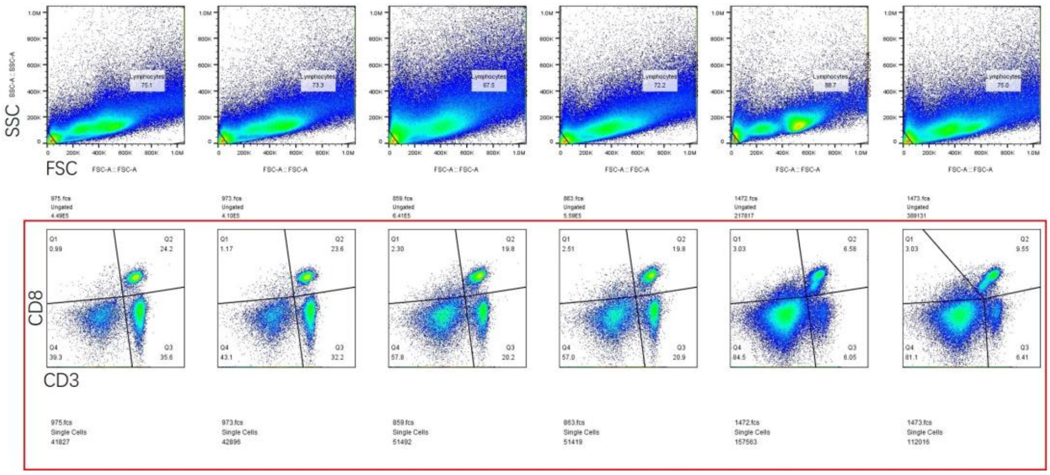

Supplementary Fig. 24. Gating strategies for Supplementary Fig. 7a.

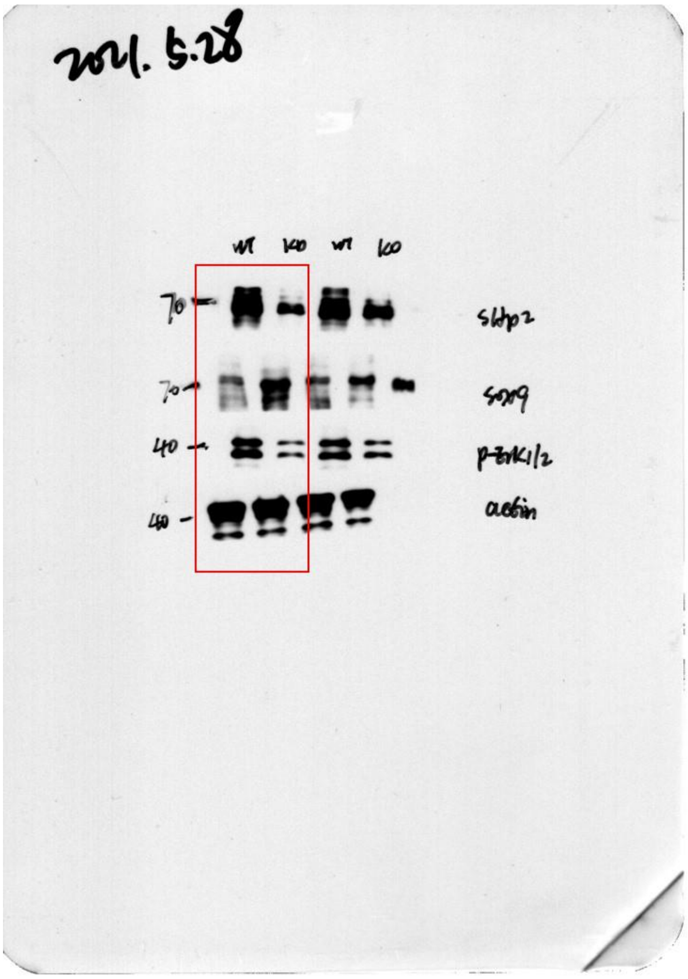

Supplementary Fig. 25. Uncropped scans of the Western blots shown in Fig. 7b.

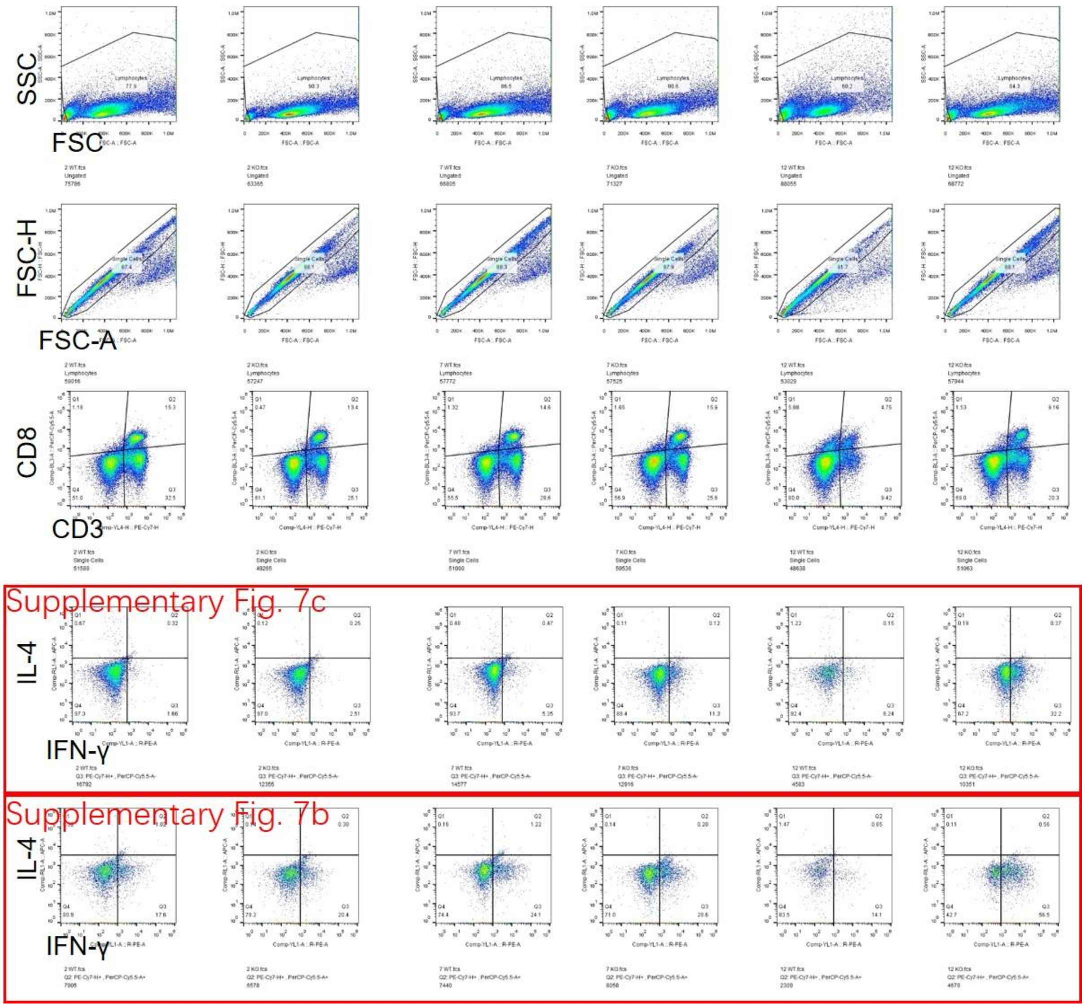

Supplementary Fig. 26. Gating strategies for Supplementary Fig. 7b and Supplementary Fig. 7c.

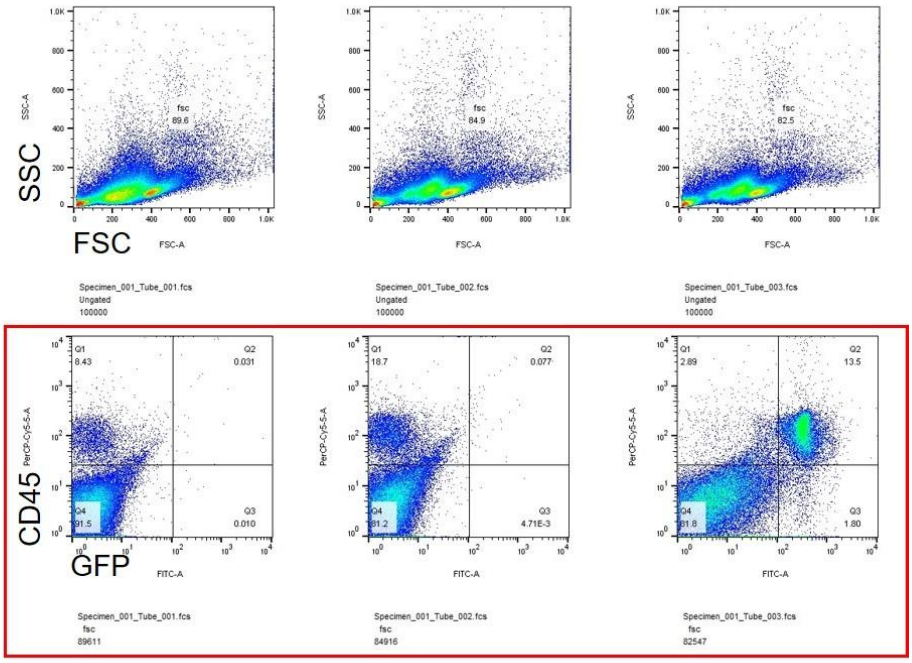

Supplementary Fig. 27. Gating strategies for Supplementary Fig. 9d.

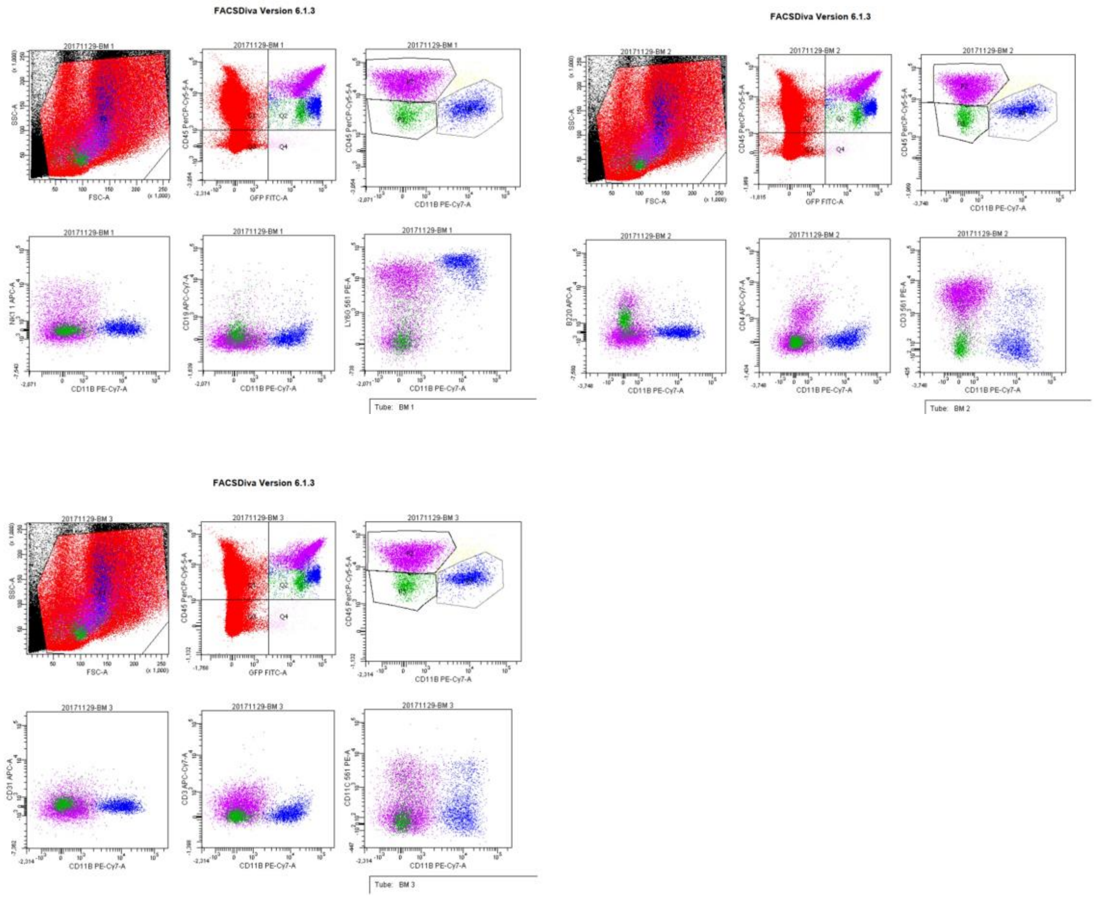

Supplementary Fig. 28. Gating strategies for Supplementary Fig. 9e.

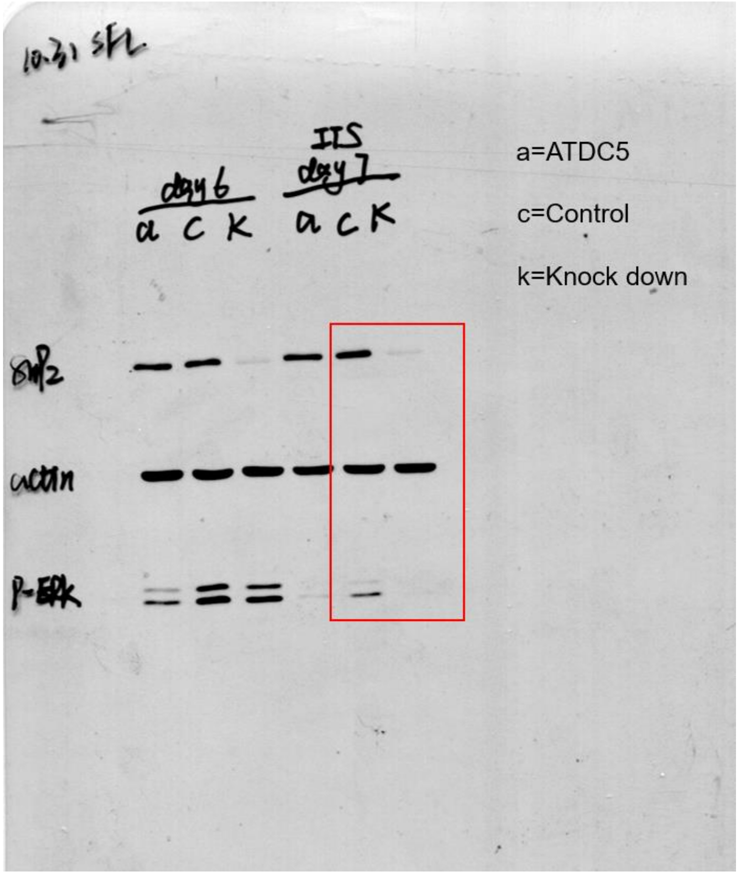

Supplementary Fig. 29. Uncropped scans of the Western blots shown in supplementary Fig. 15a.

Supplementary Table 1: Primer sequences used in RT-qPCR

Primers	Sequence (5'-3')
Mouse-*Col2a1*-F	5′- GGGAATGTCCTCTGCGATGAC
Mouse-*Col2a1*-R	5′- GAAGGGGATCTCGGGGTTG
Mouse-*Col10a1*-F	5′- TTCTGCTGCTAATGTTCTTGACC
Mouse-*Col10a1*-R	5′- GGGATGAAGTATTGTGTCTTGGG
Mouse- *Aggrecan* -F	5′- CCTGCTACTTCATCGACCCC
Mouse- *Aggrecan* -R	5′- AGATGCTGTTGACTCGAACCT
Mouse- *Sox9*-F	5′- GAGCCGGATCTGAAGAGGGA
Mouse- *Sox9*-R	5′- GCTTGACGTGTGGCTTGTTC
Mouse-*Ptpn11* -F	5′-AGAGGGAAGAGCAAATGTGTCA
Mouse- *Ptpn11*-R	5′-CTGTGTTTCCTTGTCCGACCT
Mouse-*MMP13* -F	5′-CTTCTTCTTGTTGAGCTGGACTC
Mouse-*MMP13*-R	5′-CTGTGGAGGTCACTGTAGACT
Mouse-*BMP2* -F	5′-GGGACCCGCTGTCTTCTAGT
Mouse-*BMP2*-R	5′-TCAACTCAAATTCGCTGAGGAC
Mouse-*BMP4* -F	5′-TTCCTGGTAACCGAATGCTGA
Mouse-*BMP4*-R	5′-CCTGAATCTCGGCGACTTTTT
Mouse-*BMP6* -F	5′-AGAAGCGGGAGATGCAAAAGG
Mouse-*BMP6*-R	5′-GACAGGGCGTTGTAGAGATCC
Mouse-*BMP7* -F	5′-ACGGACAGGGCTTCTCCTAC
Mouse-*BMP7*-R	5′-ATGGTGGTATCGAGGGTGGAA
Mouse-*Wnt3a* -F	5′-CTCCTCTCGGATACCTCTTAGTG
Mouse-*Wnt3a* -R	5′-GCATGATCTCCACGTAGTTCCTG
Mouse-*Wnt4* -F	5′-AGACGTGCGAGAAACTCAAAG
Mouse-*Wnt4* -R	5′-GGAACTGGTATTGGCACTCCT
Mouse-*Wnt5a* -F	5′-CAACTGGCAGGACTTTCTCAA
Mouse-*Wnt5a* -R	5′-CATCTCCGATGCCGGAACT
Mouse-*Wnt5b* -F	5′-CTGCTGACTGACGCCAACT
Mouse-*Wnt5b*-R	5′-CCTGATACAACTGACACAGCTTT
Mouse-*Wnt10b* -F	5′-GCGGGTCTCCTGTTCTTGG
Mouse-*Wnt10b*-R	5′-CCGGGAAGTTTAAGGCCCAG
Mouse-*Wnt11* -F	5′-GCTGGCACTGTCCAAGACTC
Mouse-*Wnt11*-R	5′-CTCCCGTGTACCTCTCTCCA
Mouse-*Runx2* -F	5′-ATGCTTCATTCGCCTCACAAA
Mouse-*Runx2*-R	5′-GCACTCACTGACTCGGTTGG
Mouse-*osx* -F	5′-ATGGCGTCCTCTCTGCTTG
Mouse-*osx*-R	5′-TGAAAGGTCAGCGTATGGCTT
Mouse-*OCN* -F	5′-AAGCAGGAGGGCAATAAGGT
Mouse-*OCN*-R	5′-TTTGTAGGCGGTCTTCAAGC
Mouse-*β-actin* -F	5′- GGCTGTATTCCCCTCCATCG
Mouse-*β-actin* -R	5′- CCAGTTGGTAACAATGCCATGT

Supplemental Movie 1: Bone deformations hampered the activity of mice. 2-month-old, 7-month-old and 12-month-old CD4-Ctrl mice and CD4-CKO mice were placed in the same cage and the activity of mice was recorded. Bone deformations in 7-month-old and 12-month-old CD4-CKO mice resulted in decreased activity of mice.

Supplemental Movie 2: Bone deformations occurred in CD4-CKO chimeras. Four-month-old CD4-Ctrl (WT) and CD4-CKO (KO) mice were lethally irradiated followed by transferring with bone-marrow cells. Irradiated CD4-CKO mice transferred with WT bone-marrow cells were labeled as WT-KO, and so on.12-month-old chimeras were placed in the same cage and the activity of mice was recorded. Bone deformations were detected in WT-KO chimeras and KO-KO chimeras, which resulted in decreased activity of mice.

Supplemental Movie 3: Sonidegib treatment in young mice abolished the AS-like bone disease in CD4-CKO mice. Three-week-old CD4-CKO;Rosa26-mTmG mice and CD4-Cre;Rosa26-mTmG littermates were orally gavaged every other day with Smo inhibitor, sonidegib (50 mg/kg) for 3 times. Eleven months later, the activity of CD4-Ctrl mice and CD4-CKO mice treated with sonidegib or not were recorded. Bone deformation and decreased activity were only detected in CD4-CKO mice, rather than CD4-CKO mice treated with sonidegib.

Supplemental Movie 4: Sonidegib treatment in matured mice retards AS progression in CD4-CKO mice. Seven-month-old CD4-CKO mice and CD4-Cre littermates were orally gavaged with Smo inhibitor, sonidegib (100 mg/kg) for 4 months. 11-month-old mice CD4-Ctrl mice and CD4-CKO mice treated with sonidegib or not were placed in the same cage and the activity of mice were recorded. Sonidegib treatment alleviated the bone deformations in CD4-CKO mice.
