# Supplementary material for: Targeting chondrocytes for arresting bony fusion in ankylosing spondylitis
Source: Nat Commun. 2021 Nov 11;12:6540. doi: 10.1038/s41467-021-26750-6 (PMC8585952; doi:10.1038/s41467-021-26750-6)
Supplement: Supplementary file 6 — Description of Additional Supplementary Files [file 41467_2021_26750_MOESM6_ESM.pdf]

Title: Supplemental Movie 1:

Description: Bone deformations hampered the activity of mice. 2-month-old, 7-month-old and 12-month-old CD4-Ctrl mice and CD4-CKO mice were placed in the same cage and the activity of mice was recorded. Bone deformations in 7-month-old and 12-month-old CD4-CKO mice resulted in decreased activity of mice.

Title: Supplemental Movie 2:

Description: Bone deformations occurred in CD4-CKO chimeras. Four-month-old CD4-Ctrl (WT) and CD4-CKO (KO) mice were lethally irradiated followed by transferring with bone-marrow cells. Irradiated CD4-CKO mice transferred with WT bone-marrow cells were labeled as WT-KO, and so on.12-month-old chimeras were placed in the same cage and the activity of mice was recorded. Bone deformations were detected in WT-KO chimeras and KO-KO chimeras, which resulted in decreased activity of mice.

Title: Supplemental Movie 3:

Description: Sonidegib treatment in young mice abolished the AS-like bone disease in CD4-CKO mice. Three-week-old CD4-CKO;Rosa26-mTmG mice and CD4-Cre;Rosa26-mTmG littermates were orally gavaged every other day with Smo inhibitor, sonidegib (50 mg/kg) for 3 times. Eleven months later, the activity of CD4-Ctrl mice and CD4-CKO mice treated with sonidegib or not were recorded. Bone deformation and decreased activity were only detected in CD4-CKO mice, rather than CD4-CKO mice treated with sonidegib.

Title: Supplemental Movie 4:

Description: Sonidegib treatment in matured mice retards AS progression in CD4-CKO mice. Seven-month-old CD4-CKO mice and CD4-Cre littermates were orally gavaged with Smo inhibitor, sonidegib (100 mg/kg) for 4 months. 11-month-old mice CD4-Ctrl mice and CD4-CKO mice treated with sonidegib or not were placed in the same cage and the activity of mice were recorded. Sonidegib treatment alleviated the bone deformations in CD4-CKO mice.
