# Supplementary material for: Targeting chondrocytes for arresting bony fusion in ankylosing spondylitis
Source: Nat Commun. 2021 Nov 11;12:6540. doi: 10.1038/s41467-021-26750-6 (PMC8585952; doi:10.1038/s41467-021-26750-6)
Supplement: Supplementary file 8 — Reporting Summary [file 41467_2021_26750_MOESM8_ESM.pdf]

nature portfolio

Corresponding author(s): Yang Sun

Last updated by author(s): Aug 27, 2021

Reporting Summary

Nature Portfolio wishes to improve the reproducibility of the work that we publish. This form provides structure for consistency and transparency in reporting. For further information on Nature Portfolio policies, see our Editorial Policies and the Editorial Policy Checklist.

Statistics

For all statistical analyses, confirm that the following items are present in the figure legend, table legend, main text, or Methods section.

n/a	Confirmed	
☐	☒	The exact sample size (*n*) for each experimental group/condition, given as a discrete number and unit of measurement
☐	☒	A statement on whether measurements were taken from distinct samples or whether the same sample was measured repeatedly
☐	☒	The statistical test(s) used AND whether they are one- or two-sided<br>*Only common tests should be described solely by name; describe more complex techniques in the Methods section.*
☐	☒	A description of all covariates tested
☐	☒	A description of any assumptions or corrections, such as tests of normality and adjustment for multiple comparisons
☐	☒	A full description of the statistical parameters including central tendency (e.g. means) or other basic estimates (e.g. regression coefficient) AND variation (e.g. standard deviation) or associated estimates of uncertainty (e.g. confidence intervals)
☐	☒	For null hypothesis testing, the test statistic (e.g. *F*, *t*, *r*) with confidence intervals, effect sizes, degrees of freedom and *P* value noted<br>*Give P values as exact values whenever suitable.*
☒	☐	For Bayesian analysis, information on the choice of priors and Markov chain Monte Carlo settings
☒	☐	For hierarchical and complex designs, identification of the appropriate level for tests and full reporting of outcomes
☒	☐	Estimates of effect sizes (e.g. Cohen's *d*, Pearson's *r*), indicating how they were calculated

Our web collection on statistics for biologists contains articles on many of the points above.

Software and code

Policy information about availability of computer code

Data collection	Bruker Skyscan 1276 (Bruker Preclinical Imaging), SIEMENS 1.5T, FACS Aria II (BD Biosciences), IVIS Spectrum, Zeiss LSM 880
Data analysis	1) Statistical analysis was performed with GraphPad Prism 8.2.1 (GraphPad);<br>2) MicroCT analysis with CTan 1.16.4.1 and CTvol 2.2.1 (Bruker);<br>3) FACS analysis was conducted using FACSDiva 6.1.3 (BD) or FlowJo v10 software;<br>4) MRI was performed on a 1.5T MRI scanner (Siemens);<br>5) Mouse X-Ray was performed in IVIS Spectrum;<br>6) Images were acquired by a Nikon fluorescent microscope or a Zeiss LSM 880 confocal microscope

For manuscripts utilizing custom algorithms or software that are central to the research but not yet described in published literature, software must be made available to editors and reviewers. We strongly encourage code deposition in a community repository (e.g. GitHub). See the Nature Portfolio guidelines for submitting code & software for further information.

Data

Policy information about availability of data

All manuscripts must include a data availability statement. This statement should provide the following information, where applicable:

- Accession codes, unique identifiers, or web links for publicly available datasets
- A description of any restrictions on data availability
- For clinical datasets or third party data, please ensure that the statement adheres to our policy

The data generated in this study are provided in the Supplementary Information/Source Data file. Source data are provided with this paper. Further information can be provided upon request.

Field-specific reporting

Please select the one below that is the best fit for your research. If you are not sure, read the appropriate sections before making your selection.

☒ Life sciences ☐ Behavioural & social sciences ☐ Ecological, evolutionary & environmental sciences

For a reference copy of the document with all sections, see nature.com/documents/nr-reporting-summary-flat.pdf

Life sciences study design

All studies must disclose on these points even when the disclosure is negative.

Sample size	We found bone deformation in five female CD4-CKO mice by accident. To explore the morbidity and process of bone deformation in CD4-CKO mice, we monitored ten female and male mice from birth to 12-month-old. Considering mortality of old CD4-CKO mice radiation damage, we used ten mice in each group in the experiment of bone marrow transplant and sonidegib treatment in young mice. At late stage, we chose six mice in each group in the experiment of sonidegib treatment in matured mice after we clearly know the effect of bone deformation and sonidegib on mortality of CD4-CKO mice.
Data exclusions	Owing to the deficiency of bone deformation data at initial stage, the data of five female mice found by accident were excluded from analysis.
Replication	Experimental findings were reliably reproduced. The number of independent biologic replicates is indicated in each Figure Legend.
Randomization	The bone deformation in CD4-CKO mice were compared by littermate CD4-Ctrl. The experiment of bone marrow transplant, the mice were allocated based on genotype of mice. In the experiment of sonidegib treatment, the CD4-Ctrl and CD4-CKO mice were grouped randomly to administered drugs.
Blinding	The investigators were blinded to group allocation during data collection and analysis.

Reporting for specific materials, systems and methods

We require information from authors about some types of materials, experimental systems and methods used in many studies. Here, indicate whether each material, system or method listed is relevant to your study. If you are not sure if a list item applies to your research, read the appropriate section before selecting a response.

Materials & experimental systems

n/a	Involved in the study	
☐	☒	Antibodies
☐	☒	Eukaryotic cell lines
☒	☐	Palaeontology and archaeology
☐	☒	Animals and other organisms
☐	☒	Human research participants
☒	☐	Clinical data
☒	☐	Dual use research of concern

Methods

n/a	Involved in the study	
☒	☐	ChIP-seq
☐	☒	Flow cytometry
☐	☒	MRI-based neuroimaging

Antibodies

Antibodies used	Flow cytometry antibody :<br>Anti-mouse CD3 Monoclonal Antibody (clone 17A2), (PE-Cyanine7, 25-0032-82; FITC, 11-0032-82; PE, 12-0032-82; APC-eFluor 780, 47-0032-82) eBioscience™,1:50;<br>Anti-Mouse CD8a Monoclonal Antibody (clone 53-6.7), PerCP-Cyanine5.5, eBioscience™, 45-0081-82, 1:50;<br>Anti-mouse CD4 Monoclonal Antibody (clone GK1.5), APC-Cy7, BD™, 561830, 1:50;<br>Anti-Mouse CD16/CD32 (Fcγ III/II Receptor) (clone 2.4G2), purified, BD™,553142, 1:25;

Anti-mouse IFN-γ Monoclonal Antibody (clone XMG1.2), PE, Biolegend™, 505808, 1:50;
Anti-mouse IL-2 Monoclonal Antibody (clone JES6-5H4), APC, Biolegend™, 503810, 1:50;
Anti-mouse IL-17 Monoclonal Antibody (clone TC11-18H10.1), APC, Biolegend™, 506916, 1:50;
Anti-mouse Foxp3 Monoclonal Antibody (clone MF-14), PE, eBioscience™, 12-4774-42, 1:50;
Anti-mouse CD45 Monoclonal Antibody (clone 30-F11), PerCP-Cyanine5.5, eBioscience™, 35-0451-82, 1:50;
Anti-mouse CD11b Monoclonal Antibody (clone M1/70), PE-Cyanine7, eBioscience™, 25-0112-82, 1:50;
Anti-mouse B220 Monoclonal Antibody (clone RA3-6B2), APC, eBioscience™, 17-0452-82, 1:50;
Anti-mouse CD19 Monoclonal Antibody (clone 6D5), APC-Cy7, Biolegend™,115530, 1:50;
Anti-mouse NK1.1 Monoclonal Antibody (clone PK136), APC, eBioscience™, 17-5941-82, 1:50;
Anti-mouse Ly6G Monoclonal Antibody (clone 1A8), PE, eBioscience™, 12-9668-82, 1:50;
Anti-mouse CD31 Monoclonal Antibody (clone MEC13.3) APC, Biolegend™, 102510, 1:50;
Anti-mouse CD11c Monoclonal Antibody (clone N418) PE, eBioscience™, 12-0114-82, 1:50;
Western Bolt and Immunohistochemical antibody:
anti-osteocalcin (Santa Cruz Biotechnology, sc-376835, 1:100 for IF),
anti-GFP (Cell Signaling Technology, 2555, 1:200 for IF),
anti-SOX9 (Abcam, ab76997, 1:200 for IF and WB),
anti-BrdU (Biolegend, 364102, 1:50 for IF),
anti-Cre (Cell Signaling Technology, 15036, 1:100 for IF),
anti-SHP2 (Santa Cruz Biotechnology, sc-7384, 1:100 for IF, 1:500 for WB),
anti-pSmad1/5 (Cell Signaling Technology, 9516, 1:100 for IHC, 1:1000 for WB),
anti-p-Erk1/2 (Cell Signaling Technology, 4370, 1:1000 WB),
anti-ACTIN (Abmart, M20010, 1:2000 WB)
Secondary antibodies:
Goat anti-mouse IgG2a conjugated to Alexa Fluor 594 (Invitrogen, A-21135, 1:1000),
Goat anti-mouse IgG1 conjugated to Alexa Fluor 647 (Invitrogen, A-21240, 1:1000),
Goat anti-rabbit IgG conjugated to Alexa Fluor 488 (Invitrogen, A-11008, 1:1000).

Validation

Flow cytometry antibody:
Anti-mouse CD3 Monoclonal Antibody (Cat#: 25-0032-82, 11-0032-82, 12-0032-82, 47-0032-82) reported for flow cytometry recognizing mouse CD3, CD3 Antibody, PE-Cyanine7 (25-0032-82) (thermofisher.cn), CD3 Antibody, FITC (11-0032-82) (thermofisher.cn), CD3 Antibody, PE (12-0032-82) (thermofisher.cn), CD3 Antibody, APC-eFluor® 780 (47-0032-82) (thermofisher.cn);
Anti-Mouse CD8a Monoclonal Antibody (Cat#: 45-0081-82) reported for flow cytometry recognizing mouse CD8a, CD8a Antibody, PerCP-Cyanine5.5 (45-0081-82) (thermofisher.cn);
Anti-mouse CD4 Monoclonal Antibody (Cat#:561830) reported for flow cytometry recognizing mouse CD4, APC-Cy™7 Rat Anti-Mouse CD4 (bdbiosciences.com);
Anti-Mouse CD16/CD32 (Fcγ III/II Receptor) (Cat#:553142) reported for flow cytometry recognizing mouse FcγRIII/FcγRII; Purified Rat Anti-Mouse CD16/CD32 (Mouse BD Fc Block™) (bdbiosciences.com)
Anti-mouse IFN-γ Monoclonal Antibody (Cat#:505808) reported for flow cytometry recognizing mouse IFN-γ, PE anti-mouse IFN-gamma Antibody anti-IFN-gamma - XMG1.2 (biolegend.com);
Anti-mouse IL-2 Monoclonal Antibody (Cat#:503810) reported for flow cytometry recognizing mouse IL-2, APC anti-mouse IL-2 Antibody anti-IL-2 - JES6-5H4 (biolegend.com)
Anti-mouse IL-17 Monoclonal Antibody (Cat#:506916) reported for flow cytometry recognizing mouse IL-17, APC anti-mouse IL-17A Antibody anti-IL-17A - TC11-18H10.1 (biolegend.com);
Anti-mouse Foxp3 Monoclonal Antibody (Cat#:12-4774-42) reported for flow cytometry recognizing mouse Foxp3, FOXP3 Antibody, PE (12-4774-42) (thermofisher.cn);
Anti-mouse CD45 Monoclonal Antibody (Cat#: 35-0451-82) reported for flow cytometry recognizing mouse CD45, CD45 Antibody, PE-Cyanine5.5 (35-0451-82) (thermofisher.cn);
Anti-mouse CD11b Monoclonal Antibody (Cat#:25-0112-82) reported for flow cytometry recognizing mouse CD11b, CD11b Antibody, PE-Cyanine7 (25-0112-82) (thermofisher.cn);
Anti-mouse B220 Monoclonal Antibody (Cat#:17-0452-82) reported for flow cytometry recognizing mouse B220, CD45R (B220) Antibody, APC (17-0452-82) (thermofisher.cn);
Anti-mouse CD19 Monoclonal Antibody (Cat#:115530) reported for flow cytometry recognizing mouse CD19, APC/Cyanine7 anti-mouse CD19 Antibody anti-CD19 - 6D5 (biolegend.com);
Anti-mouse NK1.1 Monoclonal Antibody (Cat#:17-5941-82) reported for flow cytometry recognizing mouse NK1.1, NK1.1 Antibody, APC (17-5941-82) (thermofisher.cn);
Anti-mouse Ly6G Monoclonal Antibody (Cat#:12-9668-82) reported for flow cytometry recognizing mouse Ly6G, Ly-6G Antibody, PE (12-9668-82) (thermofisher.cn);
Anti-mouse CD31 Monoclonal Antibody (Cat#: 102510) reported for flow cytometry recognizing mouse CD31, APC anti-mouse CD31 Antibody anti-CD31 - MEC13.3 (biolegend.com);
Anti-mouse CD11c Monoclonal Antibody (Cat#:12-0114-82) reported for flow cytometry recognizing mouse CD11c, CD11c Antibody, PE (12-0114-82) (thermofisher.cn);
Western Bolt and Immunohistochemical antibody:
anti-osteocalcin (Santa Cruz Biotechnology, sc-376835), validation reference PMID 28746340, Anti-osteocalcin Antibody (E-6) | SCBT - Santa Cruz Biotechnology;
anti-GFP (Cell Signaling Technology, 2555), validation reference PMID 32032549, GFP Antibody (cellsignal.com);
anti-SOX9 (Abcam, ab76997), validation reference PMID 2238596, Anti-SOX9 antibody [3C10] - BSA and Azide free (ab76997) | Abcam;
anti-BrdU (Biolegend, 364102), validation reference PMID 18469816, Purified anti-BrdU Antibody anti-BrdU - 3D4 (biolegend.com);
anti-Cre (Cell Signaling Technology, 15036), validation reference PMID 9288963, Cre Recombinase (D7L7L) XP® Rabbit mAb (cellsignal.com);
anti-SHP2 (Santa Cruz Biotechnology, sc-7384), validation reference PMID 34373574, Anti-SH-PTP2 Antibody (B-1) | SCBT - Santa Cruz

Biotechnology;
anti-pSmad1/5 (Cell Signaling Technology, 9516), validation reference PMID 19914168, Phospho-Smad1/5 (Ser463/465) (41D10) Rabbit mAb (cellsignal.com);
anti-p-Erk1/2 (Cell Signaling Technology, 4370), validation reference PMID 17496916, Phospho-p44/42 MAPK (Erk1/2) (Thr202/Tyr204) (D13.14.4E) XP® Rabbit mAb (cellsignal.com);
anti-ACTIN (Abmart, M20010) validation reference PMID 23749101, Actin (26F7) Mouse Antibody for PLANTs_Internal Controls- (ab-mart.com.cn);
Secondary antibodies:
Goat anti-mouse IgG2a conjugated to Alexa Fluor 594 (Invitrogen, A-21135), validation reference PMID 27853137, Goat anti-Mouse IgG2a Cross-Adsorbed, Alexa Fluor® 594 (A-21135) (thermofisher.cn)
Goat anti-mouse IgG1 conjugated to Alexa Fluor 647 (Invitrogen, A-21240), validation reference PMID 27853137, Goat anti-Mouse IgG1 Cross-Adsorbed, Alexa Fluor® 647 (A-21240) (thermofisher.cn)
Goat anti-rabbit IgG conjugated to Alexa Fluor 488 (Invitrogen, A-11008) validation reference PMID 27853137, Goat anti-Rabbit IgG (H+L) Cross-Adsorbed, Alexa Fluor® 488 (A-11008) (thermofisher.cn)

Eukaryotic cell lines

Policy information about cell lines

Cell line source(s)	The chondrogenic ATDC5 cells were kindly provided by Dr. Jian Luo (East China Normal University, Shanghai, China) (Purchased from Bluefbio, Shanghai, China).
Authentication	The cell line was not authenticated.
Mycoplasma contamination	The results of mycoplasma contaimination test of ATDC5 cell and the cell culture facility were negative.
Commonly misidentified lines (See ICLAC register)	No commonly misidentified cell lines were used.

Animals and other organisms

Policy information about studies involving animals; ARRIVE guidelines recommended for reporting animal research

Laboratory animals	The animal use and the experimental protocols were reviewed and approved by the Animal Care Committee of the Nanjing University in accordance with the Institutional Animal Care and Use Committee guidelines. Female Ptpn11f/f mice; CD4-Cre;Ptpn11f/f mice; Lck-Cre;Ptpn11f/f mice; CD4-Cre;Ptpn11f/f;Rosa26-mTmG mice were monitored from birth to 12-month-old. The mice were sacrificed at 2-month-old, 7-month-old or 12-month-old at indicated in the figure legends. 3-week-old and 7-month-old female CD4-CKO mice and the littermate control were treatment with sonidegib for three time and 4 months respectively. 4-month-old female NCG mice were transferred with CD3+ T cells isolated from 4-month-old female mice CD4-CKO mice or littermate CD4-Ctrl mice. All mice were given food and water ad libitum and housed in temperature-, moisture-, and light-controlled (12h light/dark cycle).
Wild animals	No wild animals were used in the study.
Field-collected samples	No field-collected samples were used in the study.
Ethics oversight	The animal use and the experimental protocols were reviewed and approved by the Animal Care Committee of the Nanjing University (Approval No: IACUC-2009005) in accordance with the Institutional Animal Care and Use Committee guidelines. Animal welfare and experimental procedures were carried out in accordance with the Guide for the Care and Use of Laboratory Animals (National Institutes of Health, USA) and the related ethical regulations of our university. All efforts were made to reduce the number of animals used and to minimize animal suffering.

Note that full information on the approval of the study protocol must also be provided in the manuscript.

Human research participants

Policy information about studies involving human research participants

Population characteristics	Patients with Juvenile Idiopathic Arthritis (JIA) and patients with AS. To Patients with JIA were<br>Patients with AS were from three patients (male, 23-52 years old)<br>Patients with ERA and non-ERA (13-14 years old, female and male)
Recruitment	Between 2018 and 2020, the patients with JIA that aged 13-14 years old and received treatment in Nanjing University of Chinese Medicine were recruited. The patients diagnosed with AS according to Bath Ankylosing Spondylitis Disease Activity Index (BASDAI) were recruited.
Ethics oversight	The study and protocols were carried out in accordance withthe ethical guidelines of the 1975 Declaration of Helsinki Principles, and were approved by the Ethics Institutional Review Board of Affiliated Hospital of Nanjing University of Chinese Medicine (study number 2018NL-106-02) and Children's Hospital of Nanjing Medical University (study number 202008041-1). Written informed consents were obtained from all patients.

Note that full information on the approval of the study protocol must also be provided in the manuscript.

Flow Cytometry

Plots

Confirm that:

- [x] The axis labels state the marker and fluorochrome used (e.g. CD4-FITC).
- [x] The axis scales are clearly visible. Include numbers along axes only for bottom left plot of group (a 'group' is an analysis of identical markers).
- [x] All plots are contour plots with outliers or pseudocolor plots.
- [x] A numerical value for number of cells or percentage (with statistics) is provided.

Methodology

Sample preparation	Detailed sample preparation protocol is provided in methods section of the manuscript
Instrument	FACS Aria II (BD Biosciences) and Attune NxT (Life Technologies)
Software	FACSDiva (BD) or FlowJo v10 software were used to analyze FACS data.
Cell population abundance	Quantification of cell populations are provided as total number of tissue or percentage of reference population as described in the figure and figure legends.
Gating strategy	Gating schemes were established with fluorescence-minus-one (FMO: staining with all fluorophores except one) controls

- [x] Tick this box to confirm that a figure exemplifying the gating strategy is provided in the Supplementary Information.

Magnetic resonance imaging

Experimental design

Design type	The MRI images were used to show the bone deformation of patients with AS or ERA
Design specifications	The thickness of epiphysis plate in age, sex-matched patients with ERA and no-ERA were chose to compare.
Behavioral performance measures	The patient was placed in supine position with 1.5 T high field intensity magnetic resonance (HFMRI) scanning, and body coil was used to obtain rapid spin echo at the bone lesion site.

Acquisition

Imaging type(s)	Structural diffusion
Field strength	1.5 tesla
Sequence & imaging parameters	1) Diffusion-weight imaging (DWI) with b= 0/900s/mm 3 mm thick axial slices with corresponding apparent diffusion coefficient mapping;<br>2) T1 sequences (TE 22.00 ms, TR 623.00 ms);<br>3) T2 sequences (TE 105.00 ms, TR 4710.00 ms)<br>4) T2 weighed fat saturated (T2-FS) sequence (TE 35.00 ms, TR 5740.00 ms)
Area of acquisition	Whole body scan for patients with AS, Sacroiliac joint and knee joint scan for patients with ERA

Diffusion MRI [x] Used [ ] Not used

Parameters: b value=0/900s/mm, multi shell

Preprocessing

Preprocessing software	The images of MRI results were not preprocessed and directly presents the original result.
Normalization	*If data were normalized/standardized, describe the approach(es): specify linear or non-linear and define image types used for transformation OR indicate that data were not normalized and explain rationale for lack of normalization.*
Normalization template	*Describe the template used for normalization/transformation, specifying subject space or group standardized space (e.g. original Talairach, MNI305, ICBM152) OR indicate that the data were not normalized.*
Noise and artifact removal	*Describe your procedure(s) for artifact and structured noise removal, specifying motion parameters, tissue signals and physiological signals (heart rate, respiration).*

Volume censoring | *Define your software and/or method and criteria for volume censoring, and state the extent of such censoring.*

Statistical modeling & inference

Model type and settings | There is no data statistical modeling of patients in the manuscript.

Effect(s) tested | *Define precise effect in terms of the task or stimulus conditions instead of psychological concepts and indicate whether ANOVA or factorial designs were used.*

Specify type of analysis: ☐ Whole brain ☐ ROI-based ☐ Both

Statistic type for inference (See Eklund et al. 2016) | *Specify voxel-wise or cluster-wise and report all relevant parameters for cluster-wise methods.*

Correction | *Describe the type of correction and how it is obtained for multiple comparisons (e.g. FWE, FDR, permutation or Monte Carlo).*

Models & analysis

n/a	Involved in the study
☐	☒ Functional and/or effective connectivity
☒	☐ Graph analysis
☒	☐ Multivariate modeling or predictive analysis

Functional and/or effective connectivity | The thickness of epiphysis plate in age, sex-matched patients with ERA or non-ERA were compared.
